# A TMEFF2-regulated cell cycle derived gene signature is prognostic of recurrence risk in prostate cancer

**DOI:** 10.1186/s12885-019-5592-6

**Published:** 2019-05-06

**Authors:** Constantin Georgescu, Joshua M. Corbin, Sandra Thibivilliers, Zachary D. Webb, Yan D. Zhao, Jan Koster, Kar-Ming Fung, Adam S. Asch, Jonathan D. Wren, Maria J. Ruiz-Echevarría

**Affiliations:** 1 Arthritis & Clinical Immunology Program, Division of Genomics and Data Sciences, Oklahoma Medical Research Foundation, Oklahoma City, OK USA; 2 Department of Pathology, Oklahoma University Health Sciences Center, Oklahoma City, OK USA; 3 Stephenson Cancer Center, Oklahoma City, OK USA; 4 Department of Biostatistics and Epidemiology, Oklahoma University Health Sciences Center, Oklahoma City, OK USA; 5 Amsterdam UMC, Department of Oncogenomics, University of Amsterdam, Amsterdam, The Netherlands; 6 Department of Medicine, Oklahoma University Health Sciences Center, Oklahoma City, OK USA; 7 Present address: Department of Agronomy & Horticulture, University of Nebraska–Lincoln, Lincoln, NE USA

**Keywords:** Prostate cancer, Prognostic markers, Cell cycle genes, Risk stratification, TMEFF2

## Abstract

**Background:**

The clinical behavior of prostate cancer (PCa) is variable, and while the majority of cases remain indolent, 10% of patients progress to deadly forms of the disease. Current clinical predictors used at the time of diagnosis have limitations to accurately establish progression risk. Here we describe the development of a tumor suppressor regulated, cell-cycle gene expression based prognostic signature for PCa, and validate its independent contribution to risk stratification in several radical prostatectomy (RP) patient cohorts.

**Methods:**

We used RNA interference experiments in PCa cell lines to identify a gene expression based gene signature associated with *Tmeff2,* an androgen regulated, tumor suppressor gene whose expression shows remarkable heterogeneity in PCa. Gene expression was confirmed by qRT-PCR. Correlation of the signature with disease outcome (time to recurrence) was retrospectively evaluated in four geographically different cohorts of patients that underwent RP (834 samples), using multivariate logistical regression analysis. Multivariate analyses were adjusted for standard clinicopathological variables. Performance of the signature was compared to previously described gene expression based signatures using the SigCheck software.

**Results:**

Low levels of *TMEFF2* mRNA significantly (*p* < 0.0001) correlated with reduced disease-free survival (DFS) in patients from the Memorial Sloan Kettering Cancer Center (MSKCC) dataset. We identified a panel of 11 TMEFF2 regulated cell cycle related genes (TMCC11), with strong prognostic value. TMCC11 expression was significantly associated with time to recurrence after prostatectomy in four geographically different patient cohorts (2.9 ≤ HR ≥ 4.1; *p* ≤ 0.002), served as an independent indicator of poor prognosis in the four RP cohorts (1.96 ≤ HR ≥ 4.28; *p* ≤ 0.032) and improved the prognostic value of standard clinicopathological markers. The prognostic ability of TMCC11 panel exceeded previously published oncogenic gene signatures (*p* = 0.00017).

**Conclusions:**

This study provides evidence that the TMCC11 gene signature is a robust independent prognostic marker for PCa, reveals the value of using highly heterogeneously expressed genes, like *Tmeff2*, as guides to discover prognostic indicators, and suggests the possibility that low *Tmeff2* expression marks a distinct subclass of PCa.

**Electronic supplementary material:**

The online version of this article (10.1186/s12885-019-5592-6) contains supplementary material, which is available to authorized users.

## Background

Cancer of the prostate (PCa) is the second leading cause of cancer death in male Americans. The clinical behavior of PCa is variable, and while the majority of PCa cases remain indolent, 10% of patients progress with aggressive metastatic disease and subsequent emergence of therapy-resistant PCa [1, 2]. In current practice, clinical variables including Gleason score, tumor stage, and PSA levels are used at the time of diagnosis to predict disease outcome [3, 4]. However, these prognostic factors have limitations, resulting in significant rates of overtreatment, with associated comorbidities [5–7], and undertreatment, leading to disease progression and increased risk of PCa-specific mortality [8–10].

The clinical heterogeneity of PCa reflects, in part, a remarkable genomic heterogeneity [11–18]. This suggests that disease stratification based on molecular features may be of prognostic value beyond standard clinicopathological variables, and aid in the clinical management of the disease, as is the case for other cancers, i.e. breast [19–21]. Currently, several tissue-based molecular tests offer prognostic information for patients with PCa either before or after treatment. These are based on general features of malignancy, such as the Prolaris test (initially described by Cuzick et al. [22]), which incorporates information from 31 cell-cycle related genes, or on molecular features more specific for PCa (Decipher, Oncotype DX, ProMark, and ConfirmMDx tests [23–27]). In addition, recent work has outlined the existence of several molecular subtypes of PCa [28–31]. Notably, in one of these studies, the molecular subtypes were defined by specific driver mutations or gene fusions that are essentially mutually exclusive and that are able to categorize up to 74% of the analyzed tumors [32]. If shown to correlate with clinical behavior, these molecular subtypes could prove critical for the management and treatment of the disease. However, currently their prognostic value is not fully established, and a significant fraction of primary prostate cancers in the study could not be categorized within these molecular subsets, suggesting the existence of additional relevant molecular alterations.

High levels of variability in gene expression between tumors can be useful in identifying prostate and other cancers’ risk genes [33]. We hypothesized that molecular subtypes of primary prostate cancers may exist that have gene expression patterns associated with changes in the expression of these highly variable genes. A recent report lists *TMEFF2* as one of the top 100 mRNA transcripts with the highest levels of inter-tumor variability in primary PCa tissues [34]. TMEFF2 is an androgen regulated transmembrane protein mainly restricted to brain and prostate. Our studies in PCa demonstrate a role of TMEFF2 as a tumor suppressor [35–38]. Furthermore, studies using limited numbers of clinical samples, reveal changes in the expression of *Tmeff2* with disease stage in PCa [39, 40] and gliomas [41], supporting an important role of *Tmeff2* in these diseases.

We have investigated the expression pattern of TMEFF2 in human prostate tissues and explored the potential of a TMEFF2 associated gene signature as a biomarker for disease prognosis. We report that low *TMEFF2* mRNA expression is associated with decreased disease free survival (DFS) in the MSKCC PCa dataset. Using transcriptional profiling of cell lines and publically available PCa clinical data, we have identified a low *TMEFF2* driven gene signature associated with poor clinical outcome, comprised of cell cycle related genes. This study not only provides new insights into the clinical relevance of *Tmeff2* in cancer, but also specifies a group of cell cycle related genes as prognostic and potential therapeutic targets.

## Methods

### *TMEFF2* expression data

*TMEFF2* mRNA expression in benign and malignant samples of PCa was interrogated using Oncomine Compendium of Expression Array data [42] in the following cohorts: Varambally et al. (*n* = 19; GSE3325; [43]), Vanaja et al. (*n* = 40; [44]), Grasso et al. (*n* = 122; GSE35988; [45]), and Taylor et al. (or MSKCC; *n* = 185; GSE21032; [46]).

### Validation cohorts

Four prostate cancer cohorts were used in this study to establish the prognostic value of the TMCC11 signature: MSKCC [46] (GSE21032); Cambridge [34] (GSE70768) and Stockholm [34] (GSE70769), are microarray datasets, and the TCGA PRAD (https://gdc.cancer.gov), a RNA sequencing cohort. Cancer samples for all cohorts were from RP specimens. Biochemical recurrence (MSCKK, Cambridge and Stockholm) or recurrence/progression (TCGA-PRAD) was the follow-up endpoint. Clinical, histopathological data and summary of the cohorts are listed in Table 1 and Additional file 1: Table S1.

**Table 1:** Clinical and pathological characteristics of the prostate cancer datasets used in this study

	MSKCC (Taylor et al.) *n* = 140	Cambridge (Ross-Adams et al.) *n* = 112	Stockholm (Ross-Adams et al.) *n* = 92	TCGA-PRAD (TCGA) *n* = 490
Age (years)				
Mean	58.04	62		61
Range	37.3–83.0	41–73		41–78
PSA				
Median	6.15	7.9	7.95	
< 10	104	80	56	
≥ 10	34	31	34	
Unknown	2	1	2	
Biopsy Gleason				
≤ 3 + 4	128	77	68	
≥ 4 + 3	12	27	22	
Unknown		8	2	
Surgical Gleason				
≤ 3 + 4	94	83	56	197
≥ 4 + 3	44	29	34	293
Unknown	2		2	
Extra-capsular ext.				(mri + ct scan combined)
Y	97	77	42	31
N	43	35	48	199
NA/equivocal			2	260
Positive surgical margins				
Y	33	26	42	165
N	107	86	50	311
Unknown				14
Recurrence				
Y	36	19	45	91
N	104	93	47	399
Pathology Stage				
pT2a-c	87	35	47	184
pT3a-c	46	77	42	290
pT4	7		3	10
Unknown				6

The table defines the characteristics of the samples used in this study for each of the datasets

### Mammalian cell culture and treatment

The LNCaP and 22Rv1 cell lines were purchased from American Type Culture Collection (ATCC; Manassas, VA) and cultured as recommended. Dihydrotestosterone (DHT; Sigma, Burlington,MA) was used at a concentration of 10 nM. For TMEFF2 knockdown, LNCaP and 22Rv1 cells were transduced with pLKO.1 lentiviral vectors with antisense TMEFF2 sequences shTMEFF2–0 (TRCN0000073518), shTMEFF2–1 (TRCN0000073519) and shTMEFF2–2 (TRCN0000073521). See Additional file 1: Table S7 for sequences.

### RNA extraction and RNA-Seq

LNCaP cell expressing sh_TMEFF2 or the sh_scramble control were grown for 14 days after transduction and then 24 h in hormone-depleted media before stimulation with 10 nM DHT (or ethanol as vehicle control) for 24 h prior to harvesting for RNA extraction. Three biological replicates per sample were used. Total RNA was extracted with RNeasy mini kit (Qiagen, Waltham, MA) and cDNA was synthesized with SuperScript III First-Strand synthesis system (Life Technologies Inc., Carlsbad, CA). RNA integrity and quantity was assessed using the Agilent Bioanalyzer (Agilent Technologies, Santa Clara, CA). Raw 75 bp paired-end sequences were generated from an Illumina NextSeq 500 sequencer (Illumina, San Diego, CA). Sequenced reads first underwent quality control with the FASTQC tool and then aligned to a contaminant genome to filter out reads which align to human ribosomal RNA, poly-A, poly-C, phiX virus or mitochondrial DNA sequence. The filtered reads were trimmed using Trimmomatic [47], as well as read clipping based on quality over a sliding window, retaining reads with a minimum length of 15 bp. Trimmed, filtered reads were pseudoaligned to the GRCh38 human reference transcriptome using kallisto version 0.42.3 [48], with enabled bias correction and 50 bootstrapping rounds. Expression values for 173,259 unique transcripts were measured and transcripts with an average of 5 count per million (CPM) or less across all samples were removed from further analysis. To perform differential expression analysis (LNCaP-sh_TMEFF2 vs. LNCaP-sh_scramble control), CPM values were summarized at the gene level and normalized with the *R* packages [49] and DESeq2 [50] to identify significantly differentially expressed genes (DEGs) with fold change ≥1.5 and FDR-adjusted *p*-value ≤0.05. Data deposited in NCBI GEO under accession number GSE117180.

### Real-time polymerase chain reaction (RT-PCR)

Total RNA was extracted with RNeasy mini kit and cDNA was synthesized with iScript™ Reverse Transcription Supermix for RT-qPCR (BioRad, Hercules, CA). Quantitative RT-PCR was performed using the SsoAdvanced™ Universal SYBR® Green and gene specific primers (Additional file 1: Table S7) on the Biorad CFX96™ Touch Real-Time PCR Detection System (BioRad, Hercules, CA). All RT-PCR experiments were performed under MIQE guidelines, using three biological replicates and two technical replicates.

### Western blotting

Cell lysates were prepared in RIPA buffer containing a protease inhibitor mixture and analyzed by Western blot as described before [38], using the following antibodies: TMEFF2 (HPA015587, Sigma) at a 1:1000 dilution; AR (sc-7305, Santa Cruz Biotechnology Inc., Dallas, TX) at a 1:1000 dilution; and Calnexin (ab22595; Abcam, San Francisco, CA) at a dilution of 1:4000.

### TMCC11 signature selection process

From the initial group of 25 genes nuclear genes selected as significantly upregulated (Log2 fold change ≥1.8, ≤3.1; FDR < 0.05) by DHT in the LNCaP-TMEFF2 knockdown cells, we chose the 21 top-ranking upregulated genes (Log2 fold change ≥2.0) (Additional file 1: Figure S3). We interrogated this 21 gene subset in the MSKCC dataset (*n* = 150) in cBioPortal [51, 52] and selected those genes (*n* = 11; TMCC11) whose expression was upregulated in at least 4 of those patients with low TMEFF2 mRNA expression, and that maintain a strong functional association as demonstrated using STRING [53] and IPA pathway analyses (Additional file 1: Figure S4). Two other signatures were used for SigCheck analysis. TMCC13 is a modified TMCC11 signature including two additional genes, E2F7 and GSG2 (from the TMEFF2 21 top-ranking upregulated genes; Additional file 1: Figure S3), selected based on their individual prognostic values and lack of overlap with genes from the Cuzick [22] signature. TMCC3 consist of the CDC45, NCAPG and CLSPN genes and was selected from TMCC11 as the optimal subset in predicting time to BCR in the Stockholm dataset. For this purpose, the dependence of time to BCR on the signature gene expression was modeled using GLM cox regression, and the search for the best subset relied on elastic net regularization, a standard features selection procedure implemented in the R package glmnet.

### TMCC11 signature score development

Patients were divided in two categories (high and low) based on the TMCC11 gene signature, by calculating the mean expression over all the genes in the signature for each sample. The distribution for the population was calculated, and samples were included in the high group when their mean fell within the upper tertile (above the 67th percentile) and in the low group when below the 67th percentile.

### Databases and statistics

Databases/platform used during this study: cBioportal [51, 52], Oncomine [42], the R2 genomic analysis and visualization platform (http://r2.amc.nl); the STRING database [53]; and SurvExpress [54]. The parameters used are referenced in the corresponding figure legends if applicable. For publicly available microarray or RNA-Seq expression data sets, the normalized expression data was downloaded from the Oncomine, cBioportal or R2 databases.

Hierarchical clustering of the TMCC11 signature genes (Euclidean distance with average linkage on zscore transformed expression values) on samples from the MSKCC dataset was performed in R2.

Data analysis were performed by non-parametric Wilcoxon multiple comparison test or Student t-test as indicated in figure legends. Statistical significance was defined as *P* < 0.05 unless otherwise stated. Time-to-event outcomes were evaluated using Kaplan-Meyer analysis and survival-time differences were compared using the log-rank test. Uni-, multi-variate and C-statistics were used to assess the independent effect of biomarker status on clinical outcome. Univariate hazard ratios and *p*-values were obtained using the Cox proportional hazard model. Multivariate analysis was performed using the Cox proportional hazard model. A stepwise model selection procedure coupled with Cox proportional hazard model was used to define the final model. The Harrell’s method was used to compute the concordance statistics. Covariates included in the multivariate models were: biopsy and/or surgical gleason score, PSA, pathological T- stage, positive surgical margins and/or extracapsular extension. Covariates were adjusted as follow: Gleason – High (≥4 + 3): Low (≤3 + 4); PSA – High (≥10):Low(< 10); Path Stage –High(≥T3):Low(≤T2); Positive surgical margins -Y:N; Extracapsular extension (ECE) – Y:N. These analyses were conducted using SAS 9.4 and a *p*-value of less than 0.05 or 0.01 if indicated, was deemed statistically significant.

### Gene signature analysis with SigCheck

We analyzed the prognostic potential and specificity of the TMCC11 signature using the Bioconductor package SigCheck [55]. This software allows comparison of a gene signature prognostic performance against random and known gene signatures. In a first analysis, we compared the prognostic power of the TMCC11 gene signature and 253 oncogenic signatures available from the literature. The prognostic power of a gene signature was quantified by the log-rank test *p*-value for the difference between the time to BCR in high versus low risk groups according to overall signature gene expression. Mean expression over all the genes in the signature for each sample was computed, and high versus low expression was considered as over or below the 67th percentile respectively. Log-rank *P*-values for each signature were computed using the Stockholm ( [34], GSE70769), Cambridge ( [34], GSE70768) and MSKCC ( [46], GSE21034) datasets downloaded from the GEO website. In a second analysis, we comparatively assessed the superiority of the TMCC11 and the other 253 oncogenic signatures against randomly constructed predictors. For each signature under study, 10,000 signatures of the same number of genes were selected at random and for each log-rank p-value scores of their predictive power were computed as described above. A bootstrap p-value was then determined as the proportion of random gene signatures scoring better than the original gene signature. Stockholm, Cambridge and MSKCC datasets were also used for this analysis. The code for the analysis is available upon request. See Additonal file 1 for supplementary Methods.

## Results

### Low expression of TMEFF2 is associated with advanced disease and is prognostic of clinical outcome

The previously described cell growth inhibitory function of TMEFF2 in PCa [35–37] led us to determine the relationship of *Tmeff2* expression alterations to the clinicopathologic features of PCa. We first analyzed tumor associated changes in TMEFF2 expression by immunohistochemistry in PCa tissues (Additional file 1: Figure S1A). TMEFF2 protein expression was higher in patients with localized disease as compared to non-tumor samples (not shown). However, when patients were stratified by tumor stage, TMEFF2 expression was significantly decreased in more advanced pathological stages (Additional file 1: Figure S1B).

**Fig. 1:**
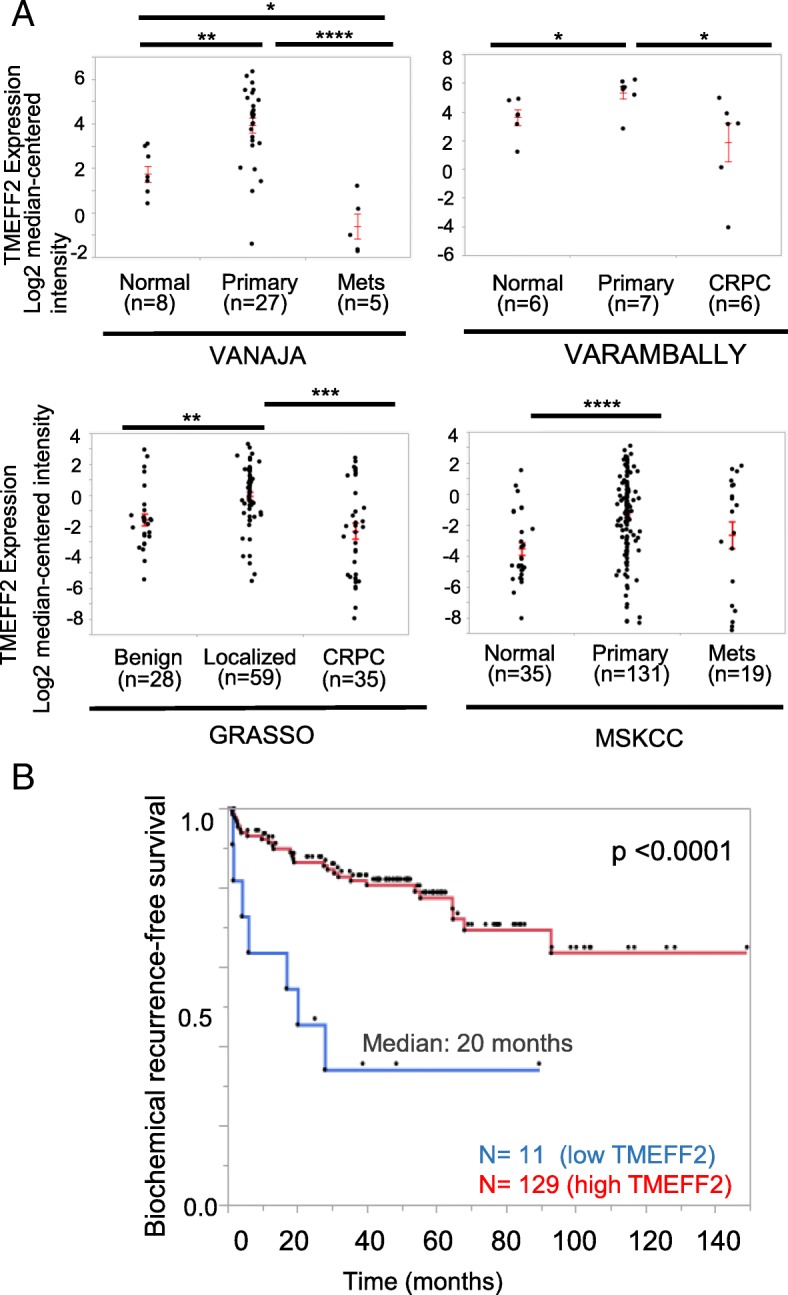
Low expression of *TMEFF2* mRNA is associated with advanced disease and clinical outcome. **a** Scatter plot showing *TMEFF2* mRNA expression levels in normal, primary and metastatic/CRPC tissue from patients from different prostate cancer cohorts. Expression levels were obtained from Oncomine and compared using a Wilcoxon multiple comparison test. **b** Kaplan-Meier analysis of biochemical-relapse free survival for patients from the MSKCC prostate cohort stratified by *TMEFF2* mRNA expression (*n* = 11; lowest expression in the cohort) vs. the rest of the patients. *P < 0.05; **P < 0.01; ***P < 0.001; ****P < 0.0001

We then used Oncomine [42] to examine alterations of *TMEFF2* mRNA expression in publically available samples from PCa patients. Expression of *TMEFF2* mRNA is significantly increased in the primary tumors of patients with PCa when compared to normal tissue, in multiple independent datasets (Fig. 1a). However, in samples from metastases and castration resistant prostate cancer (CRPC), the levels of *TMEFF2* mRNA are either unchanged or decreased compared to normal prostate, and significantly decreased (*P* < 0.05) when compared to primary tumors (Fig. 1a). These data suggest a negative correlation between *TMEFF2* mRNA expression and progression to the advanced stages of PCa.

Based on these observations, we analyzed the prognostic value of *TMEFF2* mRNA expression in the MSKCC dataset ( [46]; Table 1), a publically available human PCa dataset with clinical outcome data. Kaplan-Meier analysis demonstrated a significant (*p* < 0.0001) correlation between *TMEFF2* levels and disease progression (assessed by biochemical recurrence, BCR). Patients with the lowest *TMEFF2* mRNA expression had faster BCR (20 vs. 110 months; Fig. 1b). These findings underscore the clinical significance of *Tmeff2* in cancer.

### *TMEFF2* silencing in the LNCaP cell line increases androgen-driven expression of a group of cell-cycle related genes

**Fig. 2:**
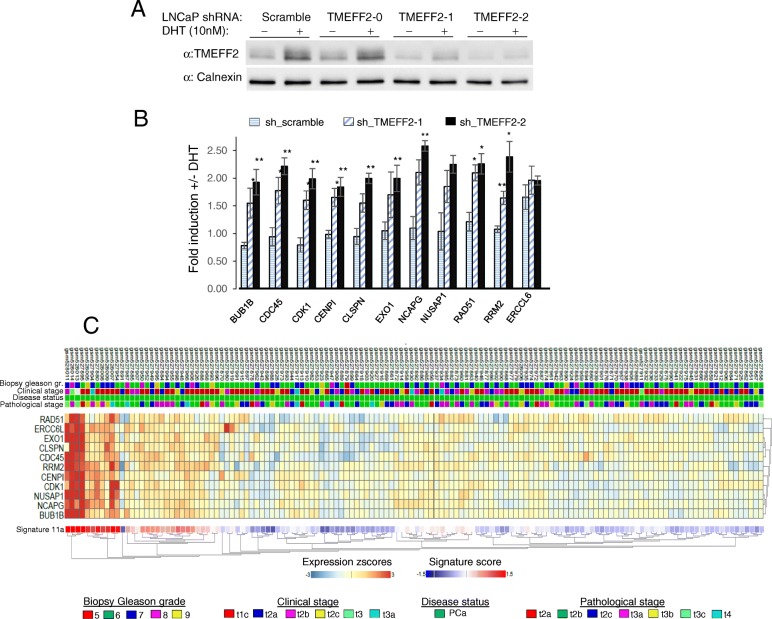
TMEFF2 silencing in PCa cells induces androgen-driven expression of cell cycle genes. **a** Western Blot analysis to determine knockdown of TMEFF2 in LNCaP cells using three different *TMEFF2* targeted shRNAs. Only sh_TMEFF2–1 and sh_TMEFF2–2 appreciably silenced *TMEFF2* expression. Note that *Tmeff2* is an androgen-regulated gene. Representative blot from > 3 repeats. **b** qRT-PCR data in the LNCaP-sh_TMEFF2 cells confirming increased expression in response to androgen stimulation of the cell cycle genes selected for the TMCC11 signature. Data is the average of 3 independent repeats and was analyzed using T-test. Error bars correspond to s.e.m. **c** Clustering analysis of TMCC11 signature genes in the MSKCC cohort. Each column corresponds to an individual patient. The status of some clinicopathological variables for each sample has been included in the figure at the top of the heatmap. *P < 0.05; **P < 0.01

*TMEFF2* is one of the top 100 mRNA transcripts with the highest levels of inter-tumor variability in patient samples from several publically available datasets ( [34] and Additional file 1: Table S1). Such heterogeneity and the fact that low *TMEFF2* mRNA expression correlates with advanced disease, suggest that it may define a molecular signature with prognostic value. To begin understanding the molecular consequences of decreased *TMEFF2* expression and its potential to define a prognostic gene signature, we conducted *TMEFF2*-targeted RNA interference experiments. Using shRNA, we silenced expression of *TMEFF2* in LNCaP cells (Fig. 2a and Additional file 1: Figures S2A and S2B), a PCa cell line that expresses high levels of *TMEFF2* mRNA and protein. Using RNA-Seq, we identified a group of 25 nuclear genes that were moderately but significantly upregulated by DHT in the context of *TMEFF2* silencing (Log2 fold change ≥1.8, ≤3.1; FDR < 0.05), as compared to control cells (transduced with scramble shRNA; Additional file 1: Figure S2C). STRING pathway analysis [53] suggests that most of these genes are functionally associated (Additional file 1: Figure S2D) and belong to the DNA replication and cell cycle gene ontology categories. All together these results suggest that *TMEFF2* silencing alters expression of androgen receptor (AR) targets, andthat previously reported TMEFF2 effects on growth [37] may be driven, in part, by TMEFF2-modulated AR-mediated expression of genes involved in cell cycle related processes (Additional file 1: Supplementary Discussion).

Out of the initial group of genes, we selected 11 (see Methods and Additional file 1: Figsure S3A and S3B) referred to as the “*TMEFF2* modulated cell cycle 11 (TMCC11)” gene signature. qRT-PCR analysis in LNCaP cells confirmed that DHT mediated induction of the TMCC11 genes was significantly increased in LNCaP cells in which *TMEFF2* expression was low compared to control cells (Fig. 2b). High expression of these genes with low *TMEFF2* expression was also seen in patients’ samples from the MSKCC dataset (Additional file 1: Figure S3C). Clustering analysis of the TMCC11 signature genes in the MSKCC dataset indicate that expression of these genes is highly correlated (Fig. 2c). These 11 genes are all tightly related to cell-cycle and DNA replication and repair processes (Additional file 1: Figure S3B). Moreover, silencing of *TMEFF2* in PCa cells affects cell cycle progression (Additional file 1: Figure S4) supporting the role of TMEFF2 in modulating expression of cell-cycle related genes (see also Additional file 1: Supplementary Discussion).

In clinical samples from the Grasso [45] and MSKCC [46] datasets, the expression of the individual genes from the TMCC11 signature is significantly increased in CRPC and metastatic disease samples when compared to normal tissue, and inversely correlated with the expression of *TMEFF2* in the same samples (Additional file 1: Figure S5A and S5B). In addition, mRNA coexpression analysis using the PCa MSKCC and PRAD TCGA datasets indicates that these genes are significantly co-expressed (Additional file 1: Figure S6).

### The TMEFF2-modulated gene signature is an independent marker of recurrence after prostatectomy in multiple clinical datasets

**Fig. 3:**
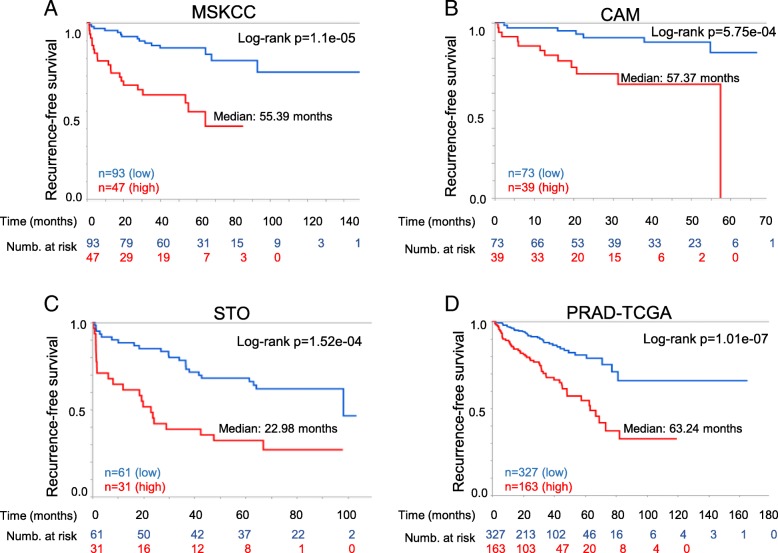
High TMCC11 expression correlates with decreased disease-free survival in several independent PCa datasets. Kaplan-Meier analysis of biochemical-relapse free survival in the MSKCC (**a**), Cambridge (**b**), Stockholm (**c**) and PRAD-TGCA (**d**) datasets. Patients were divided in two categories with the upper tertile of the TMCC11 used at the cut point. Red indicates high TMCC11 group

Based on the results suggesting that loss of TMEFF2 often predates aggressive/metastatic disease, we postulated that the TMEFF2-modulated TMCC11 gene signature could have prognostic value. We evaluated this hypothesis using BCR as the clinical endpoint in the PCa MSKCC dataset [46] (Table 1 and Additional file 1: Table S2 and Figure S7 provide information on the samples). The MSKCC dataset includes a number of prostatectomy samples from patients with wide range of times to BCR as measured by increased levels of PSA. Individually, increased expression of each of the genes comprising TMCC11 was statistically significant (*P* < 0.01) in predicting BCR (Additional file 1: Table S3; for CLSPN *p* = 0.0137). In Kaplan-Meier analyses, high expression of the TMCC11 signature was associated with a median time to progression of 55.39 months vs. greater than 150 months for patients with low expression of TMCC11 (log-rank *P* value = 1.11e-05; Fig. 3a). These results indicate that the TMCC11 signature is a powerful predictor of aggressive PCa, segregating the tumors into high and low-risk groups based on time to BCR. We obtained similar results using the SurvExpress [54] database for analysis (Additional file 1: Figure S8).

In Cox regression analyses, TMCC11 was a significant prognostic variable (*p* < 0.001) with a hazard ratio (HR) of 4.1 (Table 2). In multivariate analysis, and a model constructed using a forward stepwise selection process coupled with Cox proportional hazard, TMCC11 remained a significant prognostic variable with a HR of 2.27 and 2.35 respectively (Table 2). The final model also selected pathological T-score and surgical Gleason score as significant predictors of BCR (Table 2).

**Table 2:** Uni- and multivariate Cox regression analysis of disease recurrence in several PCa datasets

		UNIVARIATE ANALYSIS		MULTIVARIATE ANALYSIS		FINAL MODEL	
		HR (95% CI)	*p*-value	HR (95% CI)	*p*-value	HR (95% CI)	*p*-value
MSKCC	TMCC11	4.10 (2.08, 8.1)	< 0.001	2.27 (1.05,4.91)	0.038	2.35 (1.14,4.87)	0.021
	Biop. Gleason	4.26 (1.93, 9.42)	< 0.001	0.96 (0.36,2.59)	0.943		
	PSA	2.98 (1.53, 5.83)	0.001	1.66 (0.79,3.49)	0.372		
	Path Stage	4.62 (2.30,9.27)	< 0.001	4.15 (1.56,11.04)	0.459	2.99 (1.42,6.29)	0.004
	Surg. margins	2.07 (1.06, 4.06)	0.034	0.90 (0.42,1.95)	0.776		
	ECE	2.10 (0.92,4.80)	0.79	0.42 (0.12,1.29)	0.415		
	Surg. Gleason	10.56 (4.87,22.86)	< 0.001	7.92 (3.16,19.87)	< 0.001	6.78 (2.92,15.73)	< 0.001
CAM	TMCC11	4.76 (1.80, 12.59)	0.002	4.28 (1.53, 11.99)	0.006	3.53 (1.31,9.51)	0.013
	Biop. Gleason	3.25 (1.32, 8.02)	0.011	1.10 (0.25,4.93)	0.897		
	PSA	1.50 (0.57, 3.95)	0.412	2.82 (1.00,7.98)	0.050		
	Surg. Gleason	4.68 (1.88,11.63)	< 0.001	5.12 (1.07, 24.55)	0.041	4.31 (1.70,10.91)	0.002
	Surg. margins	1.64 (0.62, 4.35)	0.324	2.08 (0.76,5.71)	0.156		
	ECE	1.82 (0.60,5.48)	0.288	0.85 (0.28,2.64)	0.780		
STO	TMCC11	3.00 (1.65,5.44)	< 0.001	2.69 (1.46,6.11)	0.003	2.89 (1.56,5.36)	< 0.001
	Preop. Gleason	2.67 (1.44,4.96)	0.002	1.40 (0.66, 2.99)	0.381	2.12 (1.12,4.02)	0.021
	PSA	1.61 (0.89,2.92)	0.116	1.05 (0.56,1.98)	0.879		
	Surg. Gleason	3.62 (2.00,6.58)	< 0.001	1.77 (0.84,3.74)	0.136		
	Surg. margins	1.99 (1.10,3.59)	0.023	1.84 (0.96,3.54)	0.068		
	ECE	4.21 (2.19,8.09)	<.001	2.98 (1.46,6.11)	0.003	3.69 (1.89,7.20)	< 0.001
TCGA	TMCC11	2.94 (1.94, 4.46)	< 0.0001	1.96 (1.26,3.05)	0.003	1.96 (1.26,3.05)	0.003
	Gleason	4.08 (2.27, 7.34)	< 0.0001	2.29 (1.20, 4.38)	0.012	2.29 (1.20, 4.38)	0.012
	Path Stage	3.68 (2.07, 6.51)	< 0.0001	2.25 (1.22, 4.15)	0.010	2.25 (1.22, 4.15)	0.010

Gleason – High (≥4 + 3): Low (≤3 + 4); PSA – High (≥10):Low(< 10); Path Stage –High(≥T3):Low(≤T2); Positive surgical margins -Y:N; Extracapsular extension (ECE) – Y:N

We validated the prognostic findings in additional independent publically available datasets (see Table 1, Additional file 1: Table S2 and Fig. S7 for descriptions). Kaplan-Meier analysis of relapse free survival demonstrated that TMCC11 was a significant (log-rank *p* = 5.75e-04, *p* = 1.52e-04 and p = *P* = 1.01e-07) predictor of outcome in the Cambridge (CAM; *n* = 112; [34]), Stockholm (STO; *n* = 92; [34]) and PRAD TCGA (*n* = 490) cohorts, segregating patients with better/worse prognosis based on disease recurrence data over 60, 100 and 180 months respectively (Fig. 3b-d). Results using multivariate Cox regression analysis including expression level of the TMCC11 signature and several clinical variables, demonstrate that the TMCC11 signature is an independent predictor of recurrence after prostatectomy in these datasets (Table 2). Taken together, these data suggest that the TMCC11 signature is prognostic for risk of disease recurrence after radical prostatectomy, and has an added benefit in the context of standard clinical variables in several independent datasets.

The prognostic value of the TMCC11 signature was further evident using C-statistics (Additional file 1: Table S4). The TMCC11 signature was a significant predictor across all datasets. In the TCGA-PRAD, it performed better (C-index, 0.64; confidence interval, 0.58–0.70; *p* < 0.001) than Gleason (C-index, 0.62; confidence interval, 0.58–0.67; p < 0.001) or pathological score (C-index, 0.61; confidence interval, 0.57–0.66; p < 0.001). Moreover, in all the datasets, the TMCC11 signature significantly improved prognostic ability when combined with other clinical variables (Additional file 1: Table S4). The persistence of the interaction terms as significant effects proves that the TMCC11 predictive effectiveness might vary with the levels of the other clinical variables.

In selected patients from the MSKCC and TCGA-PRAD datasets with high pathological T (≥ T3) or Gleason (≥ 4 + 3) scores, high TMCC11 significantly stratified men at risk for disease recurrence/progression (Additional file 1: Figures S9 and S10). TMCC11 provides prognostic information in high-risk patients beyond that provided by established clinicopathological prognostic features as demonstrated using multivariate analysis (Additional file 1: Tables in Figures S9 and S10). These results suggest that TMCC11 has prognostic value in men with high-grade tumors, after RP. TMCC11 failed to stratify patients with low surgical Gleason score, however, preliminary data using the MSKCC [46] and Stockholm [34] datasets indicate that TMCC11 can stratify patients presenting with low biopsy Gleason score, suggesting that the signature may be informative for PCa management after a positive biopsy (Additional file 1: Figure S11).

### Prognostic assessment of the TMCC11 gene signature

Several gene signatures have prognostic capabilities in PCa. We therefore conducted additional tests to determine the value of the TMCC11 signature when compared to other signatures, using the Bioconductor package SigCheck [55]. This software allows comparison of a gene signature’s prognostic performance against random and known gene signatures. Initially, we analyzed the prognostic power (based on time to recurrence) of TMCC11 and other previously identified oncogenic signatures: 6 signatures for PCa [22, 25, 34, 56–58], 189 oncogenic signatures from multiple cancer types in MSigDB, and 48 breast oncogenic signatures (compiled in [59]) (*n* = 243, Table 3 and Additional file 1: Table S5). TMCC11 outperformed most signatures (Additional file 1: Table S5). Considering just the 6 PCa gene signatures, only the Cuzick (*n* = 31) signature achieved comparable performance to the TMCC11 across the three datasets for identifying patients with shorter time to biochemical relapse, and the performance depended on the dataset utilized (Table 3). Of note, 5 genes within the Cuzick set overlap with the TMCC11 set. We obtained similar results using two other TMCC11 derived signatures, TMCC13 and TMCC3 (Additional file 1: Table S5). TMCC13 is a modified form of TMCC11 including two additional genes, E2F7 and GSG2, while TMCC3 consisted of only 3 genes from the TMCC11 signature that do not overlap with the Cuzick signature. These results underscore the independent prognostic value of the genes included in the TMCC11 signature.

**Table 3:** Prognostic potential of PCa signatures

	*P*-values (compared to known PCa signatures)			*P*-values (compared to random sets of genes)			
Dataset Signature	STO	CAM	MSKCC	STO	CAM	MSKCC	Ref
CUZICK	0.00466	0.01610	2.10E-06	0.0182	0.0272	0.0000	22
TMCC11	0.00915	0.00479	0.000173	0.0305	0.0120	0.0018	This study
HES6	0.00544	0.00447	0.24900	0.0242	0.0126	0.5834	56
ROSS(100E)	0.17000	0.00720	0.06070	0.2609	0.0173	0.1388	34
IRSHAD	0.14100	0.04040	0.14500	0.2208	0.0552	0.2819	57
ONCOTYPEDX	0.05380	0.15600	0.20600	0.1126	0.1846	0.3586	25
SHARMA	0.46600	0.29700	0.60400	0.5489	0.3234	0.7237	58

Left columns: Comparative TMCC11 and known PCa signatures prognostic potential. Performance scored by log-rank test p-value of the difference on time to BCR between high and low risk groups defined by the overall gene expression signature. Right columns: Comparative analysis for TMCC11 and known PCa signatures over performance against random signatures. For each signature, 10,000 equal size signatures were generated at random and evaluated for predicting early relapse by log-rank test *p*-value. An overall bootstrap p-value score was computed as proportion of random signatures performing better than the initial signature. For both analyses, the data is sorted by first principal component of the individual rankings of the 3 columns corresponding to the Cambridge, Stockholm and MSKCC datasets. The Ross (100E) signature corresponds to the genes selected based on transcriptome profiling only. See also Additional file 1: Tables S5 and S6 for a full list with additional signatures

We then analyzed the performance of the oncogenic signatures against 10,000 signatures consisting of the same number of genes (for the specified signature) selected at random (Tables 3 and Additional file 1: Table S6). The TMCC11 signature performed in the 97th and 99th percentiles, with only 3, 1.2 and 0.18% of the random signatures demonstrating an equal or smaller *p*-value (empirical *p*-values of *p* = 0.0305, *p* = 0.012 and *p* = 0.0018) in predicting relapse in the Stockholm, Cambridge and MSKCC datasets respectively. Considering the PCa signatures, only the Cuzick (n = 31) signature achieved comparable performance to the TMCC11 across the three datasets (Table 3). TMCC11, TMCC13 and TMCC3 outperformed most of the oncogenic signatures described above (n = 243), when tested against random signatures (Additional file 1: Table S6).

## Discussion

Here, we have identified an 11-gene prognostic signature (TMCC11) for PCa progression consisting of genes associated with cell-cycle and DNA damage response. The prognostic value of this signature was confirmed on several publically available cohorts totaling 834 samples from geographically different cohorts of patients that underwent RP. TMCC11 is an independent predictor of biochemical recurrence after RP and added significant prognostic value to standard clinicopathological variables. In multivariate analysis TMCC11 was the only variable consistently predictive of disease recurrence in all of the datasets, and it significantly increased risk prediction over other clinical variables and when combined with other variables (Table 2 and Additional file 1: Table S4). Moreover, in subsets of patients with high Gleason or pathological scores, the TMCC11 signature provided a statistically significant stratification of patients identifying high and low risk groups for disease recurrence, and preliminary data suggests that TMCC11 can stratify patients that present with low biopsy or pre-operative Gleason scores. All together, these results suggest that TMCC11 may provide relevant prognostic information in several clinical scenarios and have an impact not only on the decision of whether to provide adjuvant therapy after RP, but also on treatment management after a positive biopsy.

Genomic and transcriptomic analyses have provided insight into the complexity of prostate tumors and the existence of molecular subtypes. However, the clinical applicability of these classifications has been thwarted, due in part to the highly heterogeneous nature of PCa and the difficulty of identifying additional relevant alterations that occur at low frequencies [11–18] [60]. We hypothesized that heterogeneously expressed genes can expose unidentified molecular subclasses of PCa and/or identify translationally relevant gene sets. Expression of *Tmeff2*, an androgen regulated gene, is highly variable across several different PCa datasets ( [34], Additional file 1: Table S1). Low *TMEFF2* mRNA expression significantly associated with shorter time to post-RP BCR. Although the prognostic value of low *TMEFF2* mRNA levels is uncertain, low *TMEFF2* mRNA correlates with: 1) increased androgen response of the cell cycle genes that define the TMCC11 signature in cell lines; and 2) increased mRNA levels of the same genes in samples from clinical datasets (see also Additional file 1: Supplementary Discussion). Interestingly, SPINK1 also demonstrates highly variable expression across the same datasets (Additional file 1: Table S1). SPINK1 is an androgen-regulated gene highly overexpressed in approximately 10% of PCa cases [61–63]. While the prognostic role of SPINK1 for PCa is unclear [64], it has been suggested that pathways downstream of SPINK1 may have translational and prognostic significance [64, 65]. These observations hint to highly variably expressed genes as a potential source of information with translational value.

Currently several tissue-based genomic biomarkers offer prognostic information for patients with PCa either before or after treatment [23]. The Decipher™ [24], Oncotype DX® [25] and Prolaris® [22] are commercially available panels based on measurement of gene expression changes at the RNA level. The Prolaris® panel, based on the set described in Cuzick [22], examines the expression of 31 genes involved in cell cycle progression and 5 out of the 11 genes in TMCC11 are common to this panel. We observed a similar prognostic performance for the Cuzick [22] and the TMCC11 signatures when compared against random size-matched signatures. In addition, the prognostic power (based on *p*-value) of our signature vs. Cuzick [22] was dependent on the dataset utilized, but they were similarly informative and both behaved as strong risk predictors. While these comparisons need to be verified in independent studies, TMCC11 represents a smaller and more focused distinct gene set with potentially added value in specific patient subsets. The smaller size of the TMCC11 signature (11 genes vs. 31 of Cuzick [22]) is an advantage in clinical use since smaller signatures are more amenable to testing with reduced RNA quantities (i.e. biopsy samples) or even assayed with immunohistochemistry. In addition, TMCC3, a signature consisting of three genes selected from the TMCC11 signature, that does not overlap with the Cuzick gene set, demonstrated excellent prognostic ability in SigCheck analysis. This suggests that subsets of the TMCC11 genes can be of prognostic value. Finally, the fact that our studies have independently led to the identification of a cell-cycle based signature validates the results and points to the value of using cell cycle genes as prognostic markers in PCa. See Additonal file 1 for a supplementary Discussion.

## Conclusions

Using an unconventional approach, we have identified an 11-gene signature consisting of functionally related nuclear genes with roles in DNA replication/ repair and/or cell cycle that can improve accuracy of prognosis in patients with PCa after RP in the context of current clinicopathological variables. Prognostic gene signatures containing, or based on, cell cycle gene expression changes have been identified using other approaches and different sample types. This observation not only validates our results, but also suggests that heterogeneity may lead to similar cellular consequences, providing cell cycle based signatures with rather global prognostic values. The TMCC11 signature requires further validation in multi-institutional cohorts and clinical trials. In addition, the ability of TMCC11 to provide prognostic information using biopsy samples needs to be further explored.

## Additional file

Additional file 1: A TMEFF2-regulated cell cycle derived gene signature is prognostic of recurrence risk in prostate cancer. **Figure S1.** Expression of TMEFF2 protein in prostate cancer. **Figure S2.** Androgen-induction of nuclear genes is affected by TMEFF2 silencing. **Figure S3.** Selection of the TMEFF2 modulated cell cycle (TMCC11) gene subset. **Figure S4.** Effect of TMEFF2 silencing on cell cycle progression. **Figure S5.** The TMCC11 signature genes are highly expressed in metastatic prostate cancer and clinical CRPC. **Figure S6.** The genes in the TMCC11 signature are significantly co-expressed. **Figure S7.** Distribution of the TMCC11 signature score in patients from the different datasets used in this study. **Figure S8.** High expression of TMCC11 correlates with poor prognosis in the MSKCC dataset using the SurvExpress platform for analysis. **Figure S9.** High TMCC11 expression correlates with decreased disease-free survival in subsets of patients with high pathological or surgical Gleason score in the MSKCC dataset. **Figure S10.** High TMCC11 expression correlates with decreased disease-free survival in subsets of patients with high pathological or surgical Gleason score in the PRAD-TCGA dataset. **Figure S11.** TMCC11 stratifies patients presenting with low biopsy or pre-operative Gleason score. Supplementary Methods. Supplementary Discussion. Supplementary References.** Table S1.** List of the 100 most variable expressed genes in 5 different datasets. **Table S2.** Overview of clinical datasets used in this study with expression data. **Table S3.** Summary of Kaplan-Meier analysis for DFS of the individual 11 genes corresponding to the TMCC11 signature. **Table S4.** C-statistical analysis for time to BCR comparing the performance of TMCC11 alone or in combination with other clinical variables. **Table S5.** Performance of multiple oncogenic signatures on predicting relapse. **Table S6.** Comparison of the prognostic potential for relapse of multiple oncogenic signatures against random sets of genes. **Table S7.** Primers and TMEFF2 shRNA targets used in this study (DOCX 3760 kb)

## Abbreviations

AR: Androgen receptor
BCR: Biochemical recurrence
CPM: Counts per million
CRPC: Castration resistant prostate cancer
DEG: Differentially expressed gene
DFS: Disease free survival
FDR: False discovery rate
PCa: Prostate cancer
PSA: Prostate specific antigen
qRT-PCR: Quantitative reverse-transcription polymerase chain reaction
RP: Radical prostatectomy
TMEFF2: Transmembrane protein with EGF like and two follistatin domains 2

## References

[1] Siegel RL, Miller KD, Jemal A (2015). Cancer statistics, 2015. CA Cancer J Clin.

[2] Shtivelman E, Beer TM, Evans CP (2014). Molecular pathways and targets in prostate cancer. Oncotarget..

[3] Humphrey PA (2004). Gleason grading and prognostic factors in carcinoma of the prostate. Mod Pathol.

[4] Joniau S, Briganti A, Gontero P, Gandaglia G, Tosco L, Fieuws S (2015). Stratification of high-risk prostate cancer into prognostic categories: a European multi-institutional study. Eur Urol.

[5] Draisma G, Etzioni R, Tsodikov A, Mariotto A, Wever E, Gulati R (2009). Lead time and overdiagnosis in prostate-specific antigen screening: importance of methods and context. J Natl Cancer Inst.

[6] Hong SK, Vertosick E, Sjoberg DD, Scardino PT, Eastham JA (2014). Insignificant disease among men with intermediate-risk prostate cancer. World J Urol.

[7] Loeb S, Bjurlin MA, Nicholson J, Tammela TL, Penson DF, Carter HB (2014). Overdiagnosis and overtreatment of prostate Cancer. Eur Urol.

[8] Shao YH, Demissie K, Shih W, Mehta AR, Stein MN, Roberts CB (2009). Contemporary risk profile of prostate cancer in the United States. J Natl Cancer Inst.

[9] Schröder FH, Hugosson J, Roobol MJ, Tammela TLJ, Ciatto S, Nelen V (2012). Prostate-Cancer mortality at 11 years of follow-up. N Engl J Med.

[10] Aizer AA, Chen MH, Hattangadi J, D'Amico AV (2014). Initial management of prostate-specific antigen-detected, low-risk prostate cancer and the risk of death from prostate cancer. BJU Int.

[11] Ruijter ET, van de Kaa CA, Schalken JA, Debruyne FM, Ruiter DJ (1996). Histological grade heterogeneity in multifocal prostate cancer. Biological and clinical implications. J Pathol.

[12] Boutros PC, Fraser M, Harding NJ, de Borja R, Trudel D, Lalonde E (2015). Spatial genomic heterogeneity within localized, multifocal prostate cancer. Nat Genet.

[13] Cyll K, Ersvær E, Vlatkovic L, Pradhan M, Kildal W, Avranden Kjær M (2017). Tumour heterogeneity poses a significant challenge to cancer biomarker research. Br J Cancer.

[14] Boutros PC, Fraser M, van der Kwast T, Bristow RG (2016). Clonality of localized and metastatic prostate cancer. Curr Opin Urol.

[15] Tosoian Jeffrey J., Antonarakis Emmanuel S. (2017). Molecular heterogeneity of localized prostate cancer: more different than alike. Translational Cancer Research.

[16] Shoag J, Barbieri C (2016). Clinical variability and molecular heterogeneity in prostate cancer. Asian J Androl.

[17] Cooper CS, Eeles R, Wedge DC, Van Loo P, Gundem G, Alexandrov LB (2015). Analysis of the genetic phylogeny of multifocal prostate cancer identifies multiple independent clonal expansions in neoplastic and morphologically normal prostate tissue. Nat Genet.

[18] Gundem G, Van Loo P, Kremeyer B, Alexandrov LB, Tubio JMC, Papaemmanuil E (2015). The evolutionary history of lethal metastatic prostate cancer. Nature..

[19] Guiu S, Michiels S, Andre F, Cortes J, Denkert C, Di Leo A (2012). Molecular subclasses of breast cancer: how do we define them? The IMPAKT 2012 working group statement. Ann Oncol.

[20] Bertucci F, Finetti P, Cervera N, Maraninchi D, Viens P, Birnbaum D (2006). Gene expression profiling and clinical outcome in breast cancer. Omics.

[21] Parker JS, Mullins M, Cheang MCU, Leung S, Voduc D, Vickery T (2009). Supervised risk predictor of breast Cancer based on intrinsic subtypes. J Clin Oncol.

[22] Cuzick J, Swanson GP, Fisher G, Brothman AR, Berney DM, Reid JE (2011). Prognostic value of an RNA expression signature derived from cell cycle proliferation genes in patients with prostate cancer: a retrospective study. Lancet Oncol.

[23] Moschini M, Spahn M, Mattei A, Cheville J, Karnes RJ (2016). Incorporation of tissue-based genomic biomarkers into localized prostate cancer clinics. BMC Med.

[24] Erho N, Crisan A, Vergara IA, Mitra AP, Ghadessi M, Buerki C (2013). Discovery and validation of a prostate cancer genomic classifier that predicts early metastasis following radical prostatectomy. PLoS One.

[25] Klein EA, Cooperberg MR, Magi-Galluzzi C, Simko JP, Falzarano SM, Maddala T (2014). A 17-gene assay to predict prostate cancer aggressiveness in the context of Gleason grade heterogeneity, tumor multifocality, and biopsy undersampling. Eur Urol.

[26] Blume-Jensen P, Berman DM, Rimm DL, Shipitsin M, Putzi M, Nifong TP (2015). Development and clinical validation of an in situ biopsy-based multimarker assay for risk stratification in prostate cancer. Clin Cancer Res.

[27] Partin AW, Van Neste L, Klein EA, Marks LS, Gee JR, Troyer DA (2014). Clinical validation of an epigenetic assay to predict negative histopathological results in repeat prostate biopsies. J Urol.

[28] Kaffenberger SD, Barbieri CE (2016). Molecular subtyping of prostate cancer. Curr Opin Urol.

[29] Schoenborn JR, Nelson P, Fang M (2013). Genomic profiling defines subtypes of prostate cancer with the potential for therapeutic stratification. Clin Cancer Res.

[30] Demichelis F, Garraway LA, Rubin MA (2013). Molecular archeology: unearthing androgen-induced structural rearrangements in prostate cancer genomes. Cancer Cell.

[31] Lee D, Fontugne J, Gumpeni N, Park K, MacDonald TY, Robinson BD (2017). Molecular alterations in prostate cancer and association with MRI features. Prostate Cancer Prostatic Dis.

[32] Aea A (2015). The molecular taxonomy of primary prostate Cancer. Cell..

[33] Gorlov IP, Yang J-Y, Byun J, Logothetis C, Gorlova OY, Do K-A (2014). How to get the most from microarray data: advice from reverse genomics. BMC Genomics.

[34] Ross-Adams H, Lamb AD, Dunning MJ, Halim S, Lindberg J, Massie CM (2015). Integration of copy number and transcriptomics provides risk stratification in prostate cancer: a discovery and validation cohort study. EBioMedicine..

[35] Chen X, Corbin JM, Tipton GJ, Yang LV, Asch AS, Ruiz-Echevarria MJ (2014). The TMEFF2 tumor suppressor modulates integrin expression, RhoA activation and migration of prostate cancer cells. Biochim Biophys Acta.

[36] Chen X, Overcash R, Green T, Hoffman D, Asch AS, Ruiz-Echevarria MJ (2011). The tumor suppressor activity of the transmembrane protein with epidermal growth factor and two follistatin motifs 2 (TMEFF2) correlates with its ability to modulate sarcosine levels. J Biol Chem.

[37] Corbin JM, Overcash RF, Wren JD, Coburn A, Tipton GJ, Ezzell JA (2016). Analysis of TMEFF2 allografts and transgenic mouse models reveals roles in prostate regeneration and cancer. Prostate..

[38] Green T, Chen X, Ryan S, Asch AS, Ruiz-Echevarria MJ (2013). TMEFF2 and SARDH cooperate to modulate one-carbon metabolism and invasion of prostate cancer cells. Prostate..

[39] Afar DE, Bhaskar V, Ibsen E, Breinberg D, Henshall SM, Kench JG (2004). Preclinical validation of anti-TMEFF2-auristatin E-conjugated antibodies in the treatment of prostate cancer. Mol Cancer Ther.

[40] Glynne-Jones E, Harper ME, Seery LT, James R, Anglin I, Morgan HE (2001). TENB2, a proteoglycan identified in prostate cancer that is associated with disease progression and androgen independence. Int J Cancer.

[41] Lin K, Taylor JR, Wu TD, Gutierrez J, Elliott JM, Vernes J-M (2011). TMEFF2 is a PDGF-AA binding protein with methylation-associated gene silencing in multiple Cancer types including glioma. PLoS One.

[42] Rhodes DR, Yu J, Shanker K, Deshpande N, Varambally R, Ghosh D (2004). ONCOMINE: a cancer microarray database and integrated data-mining platform. Neoplasia (New York, NY).

[43] Varambally S, Yu J, Laxman B, Rhodes DR, Mehra R, Tomlins SA (2005). Integrative genomic and proteomic analysis of prostate cancer reveals signatures of metastatic progression. Cancer Cell.

[44] Vanaja DK, Cheville JC, Iturria SJ, Young CY (2003). Transcriptional silencing of zinc finger protein 185 identified by expression profiling is associated with prostate cancer progression. Cancer Res.

[45] Grasso CS, Wu YM, Robinson DR, Cao X, Dhanasekaran SM, Khan AP (2012). The mutational landscape of lethal castration-resistant prostate cancer. Nature..

[46] Taylor BS, Schultz N, Hieronymus H, Gopalan A, Xiao Y, Carver BS (2010). Integrative genomic profiling of human prostate cancer. Cancer Cell.

[47] Bolger AM, Lohse M, Usadel B (2014). Trimmomatic: a flexible trimmer for Illumina sequence data. Bioinformatics..

[48] Bray NL, Pimentel H, Melsted P, Pachter L (2016). Near-optimal probabilistic RNA-seq quantification. Nat Biotechnol.

[49] Soneson C, Love MI, Robinson MD (2015). Differential analyses for RNA-seq: transcript-level estimates improve gene-level inferences. F1000Res..

[50] Love MI, Huber W, Anders S (2014). Moderated estimation of fold change and dispersion for RNA-seq data with DESeq2. Genome Biol.

[51] Cerami E, Gao J, Dogrusoz U, Gross BE, Sumer SO, Aksoy BA (2012). The cBio Cancer genomics portal: an open platform for exploring multidimensional Cancer genomics data. Cancer Discov.

[52] Gao J, Aksoy BA, Dogrusoz U, Dresdner G, Gross B, Sumer SO (2013). Integrative analysis of complex Cancer genomics and clinical profiles using the cBioPortal. Sci Signal.

[53] Szklarczyk D, Morris JH, Cook H, Kuhn M, Wyder S, Simonovic M (2017). The STRING database in 2017: quality-controlled protein-protein association networks, made broadly accessible. Nucleic Acids Res.

[54] Aguirre-Gamboa R, Gomez-Rueda H, Martínez-Ledesma E, Martínez-Torteya A, Chacolla-Huaringa R, Rodriguez-Barrientos A (2013). SurvExpress: an online biomarker validation tool and database for Cancer gene expression data using survival analysis. PLoS One.

[55] Stark R, Norder J. SigCheck: Check a gene signature's prognostic performance against random signatures, known signatures, and permuted data. 2016.

[56] Ramos-Montoya A, Lamb AD, Russell R, Carroll T, Jurmeister S, Galeano-Dalmau N (2014). HES6 drives a critical AR transcriptional programme to induce castration-resistant prostate cancer through activation of an E2F1-mediated cell cycle network. EMBO Mol Med.

[57] Irshad S, Bansal M, Castillo-Martin M, Zheng T, Aytes A, Wenske S (2013). A molecular signature predictive of indolent prostate Cancer. Sci Transl Med.

[58] Sharma Naomi L., Massie Charlie E., Ramos-Montoya Antonio, Zecchini Vincent, Scott Helen E., Lamb Alastair D., MacArthur Stewart, Stark Rory, Warren Anne Y., Mills Ian G., Neal David E. (2013). The Androgen Receptor Induces a Distinct Transcriptional Program in Castration-Resistant Prostate Cancer in Man. Cancer Cell.

[59] Venet D, Dumont JE, Detours V (2011). Most random gene expression signatures are significantly associated with breast Cancer outcome. PLoS Comput Biol.

[60] Armenia J, Wankowicz SAM, Liu D, Gao J, Kundra R, Reznik E, et al. The long tail of oncogenic drivers in prostate cancer. Nat Genet. 2018. 10.1038/s41588-018-0078-z PMC6107367 29610475

[61] Paju A, Hotakainen K, Cao Y, Laurila T, Gadaleanu V, Hemminki A (2007). Increased expression of tumor-associated trypsin inhibitor, TATI, in prostate cancer and in androgen-independent 22Rv1 cells. Eur Urol.

[62] Stenman UH (2011). SPINK1: a new therapeutic target in cancer?. Clin Chem.

[63] Tomlins SA, Rhodes DR, Yu J, Varambally S, Mehra R, Perner S (2008). The role of SPINK1 in ETS rearrangement-negative prostate cancers. Cancer Cell.

[64] Flavin R, Pettersson A, Hendrickson WK, Fiorentino M, Finn S, Kunz L (2014). SPINK1 protein expression and prostate cancer progression. Clin Cancer Res.

[65] Ateeq B, Tomlins SA, Laxman B, Asangani IA, Cao Q, Cao X (2011). Therapeutic targeting of SPINK1-positive prostate cancer. Sci Transl Med.

