# Supplementary material for: A TMEFF2-regulated cell cycle derived gene signature is prognostic of recurrence risk in prostate cancer
Source: BMC Cancer. 2019 May 6;19:423. doi: 10.1186/s12885-019-5592-6 (PMC6503380; doi:10.1186/s12885-019-5592-6)
Supplement: Supplementary file 1 — A TMEFF2-regulated cell cycle derived gene signature is prognostic of recurrence risk in prostate cancer. Figure S1. Expression of TMEFF2 protein in prostate cancer. Figure S2. Androgen-induction of nuclear genes is affected by TMEFF2 silencing. Figure S3. Selection of the TMEFF2 modulated cell cycle (TMCC11) gene subset. Figure S4. Effect of TMEFF2 silencing on cell cycle progression. Figure S5. The TMCC11 signature genes are highly expressed in metastatic prostate cancer and clinical CRPC. Figure S6. The genes in the TMCC11 signature are significantly co-expressed. Figure S7. Distribution of the TMCC11 signature score in patients from the different datasets used in this study. Figure S8. High expression of TMCC11 correlates with poor prognosis in the MSKCC dataset using the SurvExpress platform for analysis. Figure S9. High TMCC11 expression correlates with decreased disease-free survival in subsets of patients with high pathological or surgical Gleason score in the MSKCC dataset. Figure S10. High TMCC11 expression correlates with decreased disease-free survival in subsets of patients with high pathological or surgical Gleason score in the PRAD-TCGA dataset. Figure S11. TMCC11 stratifies patients presenting with low biopsy or pre-operative Gleason score. Supplementary Methods. Supplementary Discussion. Supplementary References. Table S1. List of the 100 most variable expressed genes in 5 different datasets. Table S2. Overview of clinical datasets used in this study with expression data. Table S3. Summary of Kaplan-Meier analysis for DFS of the individual 11 genes corresponding to the TMCC11 signature. Table S4. C-statistical analysis for time to BCR comparing the performance of TMCC11 alone or in combination with other clinical variables. Table S5. Performance of multiple oncogenic signatures on predicting relapse. Table S6. Comparison of the prognostic potential for relapse of multiple oncogenic signatures against random sets of genes. Table S7. Primers and TMEFF [file 12885_2019_5592_MOESM1_ESM.docx]

SUPPLEMENTARY MATERIALS FOR:

A TMEFF2-REGULATED CELL CYCLE GENE SIGNATURE IS PROGNOSTIC OF RECURRENCE RISK IN PROSTATE CANCER

Georgescu et al.

This PDF file includes:

Supplementary Methods

Supplementary Discussion

Supplementary References

Supplementary Figures S1-S11

Supplementary Tables S1-S7

**SUPPLEMENTARY METHODS**

**Patient samples for TMEFF2 immunohistochemistry**

Radical prostatectomy histopathology records from the Department of Pathology at the University of Oklahoma Health Sciences Center (OUSHC) were retrospectively examined. De-identified, archived, PCa specimens from Oklahoma patients with localized and metastatic disease, collected between 2005 and 2015, were obtained (n=39). Only pathological T-score and Gleason score clinical data were evaluated. All the other clinical samples were from publically available datasets described below.

**Institutional Review Board Approval**

This study was approved by the institutional review board of the University of Oklahoma Health Sciences Center. The institutional review board issued an expedited review and waived the need for written consent for this study because only archival, de-identified materials were used.

**Histology and Immunohistochemistry**

Paraffin embedded prostate biopsies were selected and 5-μm sections were prepared for hematoxilin & eosin (H&E) staining and immunohistochemistry (IHC). IHC was performed according to manufacturer’s protocol using Leica Bond-III^TM^ Polymer Refine Detection system (DS 9800). Briefly, the slides with the sections were deparaffinized and rehydrated in an automated Multistainer (Leica ST5020, Leica Biosystems, Buffalo Grove, IL) and transferred to the Leica Bond-III^TM^ for antigen retrieval at 100°C (20-40 minutes). Endogenous peroxidase was blocked using peroxidase-blocking reagent, followed by incubation with the TMEFF2 antibody (Sigma; 1:150 dilution) for 60 minutes and then post-primary IgG-linker and/or Poly-HRP IgG reagents**.** For image analysis, the slides were scanned into digital images using an Aperio CS scanner (Leica Biosystems). Positive stain was quantified in the selected areas using the Aperio positive pixel count algorithm and areas of positive stain in each sample added together. A correlation between TMEFF2 positivity and pathological T-score was established.

**Cell Cycle analysis**

For cell cycle analysis, cells were synchronized by treatment with Aphidicolin (Sigma, Burlington, MA) at a final concentration of 2μg/ml for 24hours. Flow cytometric analysis was performed as described before (1) from cells released from the drug at the indicated timepoints, using a FACSCalibur device (BD Biosciences, San Jose, CA) and the ModFit LT V4.1.7 software.

**SUPPLEMENTARY DISCUSSION**

**Background:** Clinical progression to aggressive PCa and ultimately to CRPC, is the cause of death for most patients dying from this disease. In patients undergoing radical prostatectomy (RP), risk stratification guides the use of adjuvant therapy and follow-up. However, current clinicopathological variables provide limited prognostic information and, while not all the patients presenting with high grade tumors relapse after RP, some that do not present with adverse characteristics do (2-9). Therefore, adjuvant therapy subjects many patients to unnecessary treatment and the potential for side effects. Improvements to the prediction of the risk of recurrence after curative treatment are therefore necessary for disease management. A second major obstacle on the clinical management of PCa relates to overdiagnosis and overtreatment of patients with newly diagnosed disease. Many of these patients will present with indolent disease that could be managed by active surveillance, avoiding surgical treatment and potential complications derived from it (10-12).

Here we described the identification of an 11-gene prognostic signature (TMCC11) for PCa progression consisting of genes associated with cell-cycle and DNA damage response, and presented data suggesting that TMCC11 may provide relevant prognostic information in several clinical scenarios and have an impact not only on the decision of whether to provide adjuvant therapy after RP, but also on treatment management after a positive biopsy.

**TMEFF2, androgen receptor and cell cycle genes:** We hypothesized that heterogeneously expressed genes can expose unidentified molecular subclasses and may define prognostic signatures. To test this we selected *Tmeff2,* an androgen regulated gene (13, 14) and one of the top 100 transcripts with the highest levels of inter-tumor variability in primary PCa tissues (this study and (15)). Overexpression of TMEFF2 has been shown to inhibit proliferation of the AR negative PCa cell lines, DU145 and PC3, which do not express TMEFF2 (13). Likewise, our published data indicates that TMEFF2 functions as a tumor suppressor in PCa by inhibiting allograft growth and cell motility (1, 16-18). Consistent with this, here we report an inverse correlation between *TMEFF2* mRNA expression and high-grade localized prostate cancer as well as metastatic lesions. This also correlates with a report of loss of *Tmeff2* expression due to increased promoter methylation in metastatic PCa (19). Low *TMEFF2* expression significantly associated with shorter time to post-RP BCR, however the prognostic value of low *TMEFF2* mRNA levels was limited by sample size, and additional experiments are needed to characterize this role of TMEFF2. Interestingly, *TMEFF2* mRNA levels are increased in the lower grade primary tumors. This pattern of expression, high in primary and low in metastatic disease, has been reported for other androgen-regulated genes (20).

Importantly, we identified TMCC11 as a signature consisting of cell cycle genes that are upregulated in patients with low *TMEFF2* mRNA levels. In experiments with cell lines, TMEFF2 silencing promoted increased androgen response of these genes, indicating AR involvement in this effect. The role of the AR in controlling cell-cycle progression is well documented and complex. It regulates gene networks that are retained throughout the cycle, as well as some that are specific for a cell cycle phase (21), and reciprocally, several elements of the cell cycle machinery modulate AR activity, contributing to the cell cycle phase specific function of the AR (21-25). The results presented here also point to a role of TMEFF2 in modulating AR activity and ultimately the expression of cell cycle genes. Most of the genes from the TMCC11 signature are shown to be upregulated on the S and/or G2-M phases of the cell cycle and correspond to genes expressed in aggressive forms of the disease (26, 27). The effect of TMEFF2 on AR could therefore contribute to its tumor suppressor function. Besides cell cycle, other major pathways affected by TMEFF2 silencing (with respect to the number of functionally related genes affected) are associated with ER-unfolded protein response and lipid metabolism, pathways that are also affected by AR function and have important roles in PCa. Finally, our unpublished data indicates that androgen induction of some AR-modulated genes important for metastases (i.e. NRDG1) are affected by TMEFF2 silencing, suggesting that TMEFF2 may also modulate other AR-responsive genes with roles in epithelial-to-mesenchymal transition (EMT), migration and metastatic processes, and in this way play a role in the clinical progression in PCa.

**SUPPLEMENTARY REFERENCES (for supplementary discussion only)**

1. Chen X, Overcash R, Green T, Hoffman D, Asch AS, Ruiz-Echevarria MJ. The tumor suppressor activity of the transmembrane protein with epidermal growth factor and two follistatin motifs 2 (TMEFF2) correlates with its ability to modulate sarcosine levels. J Biol Chem. 2011;286(18):16091-100.

2. Roehl KA, Han M, Ramos CG, Antenor JA, Catalona WJ. Cancer progression and survival rates following anatomical radical retropubic prostatectomy in 3,478 consecutive patients: long-term results. The Journal of urology. 2004;172(3):910-4.

3. Freedland SJ, Humphreys EB, Mangold LA, Eisenberger M, Dorey FJ, Walsh PC, et al. Risk of prostate cancer-specific mortality following biochemical recurrence after radical prostatectomy. Jama. 2005;294(4):433-9.

4. Antonarakis ES, Feng Z, Trock BJ, Humphreys EB, Carducci MA, Partin AW, et al. The natural history of metastatic progression in men with prostate-specific antigen recurrence after radical prostatectomy: long-term follow-up. BJU international. 2012;109(1):32-9.

5. Paller CJ, Antonarakis ES. Management of Biochemically Recurrent Prostate Cancer After Local Therapy: Evolving Standards of Care and New Directions. Clinical advances in hematology & oncology : H&O. 2013;11(1):14-23.

6. Amling CL, Blute ML, Bergstralh EJ, Seay TM, Slezak J, Zincke H. Long-term hazard of progression after radical prostatectomy for clinically localized prostate cancer: continued risk of biochemical failure after 5 years. The Journal of urology. 2000;164(1):101-5.

7. Han M, Partin AW, Pound CR, Epstein JI, Walsh PC. Long-term biochemical disease-free and cancer-specific survival following anatomic radical retropubic prostatectomy. The 15-year Johns Hopkins experience. The Urologic clinics of North America. 2001;28(3):555-65.

8. Hull GW, Rabbani F, Abbas F, Wheeler TM, Kattan MW, Scardino PT. Cancer control with radical prostatectomy alone in 1,000 consecutive patients. The Journal of urology. 2002;167(2 Pt 1):528-34.

9. Psutka SP, Feldman AS, Rodin D, Olumi AF, Wu CL, McDougal WS. Men with organ-confined prostate cancer and positive surgical margins develop biochemical failure at a similar rate to men with extracapsular extension. Urology. 2011;78(1):121-5.

10. Loeb S, Bjurlin M, Nicholson J, Tammela TL, Penson D, Carter HB, et al. Overdiagnosis and Overtreatment of Prostate Cancer. European urology. 2014;65(6):1046-55.

11. Draisma G, Etzioni R, Tsodikov A, Mariotto A, Wever E, Gulati R, et al. Lead time and overdiagnosis in prostate-specific antigen screening: importance of methods and context. J Natl Cancer Inst. 2009;101(6):374-83.

12. Klotz L. Prostate cancer overdiagnosis and overtreatment. Current opinion in endocrinology, diabetes, and obesity. 2013;20(3):204-9.

13. Gery S, Sawyers CL, Agus DB, Said JW, Koeffler HP. TMEFF2 is an androgen-regulated gene exhibiting antiproliferative effects in prostate cancer cells. Oncogene. 2002;21(31):4739-46.

14. Overcash RF, Chappell VA, Green T, Geyer CB, Asch AS, Ruiz-Echevarría MJ. Androgen Signaling Promotes Translation of TMEFF2 in Prostate Cancer Cells via Phosphorylation of the α Subunit of the Translation Initiation Factor 2. PloS one. 2013;8(2):e55257.

15. Ross-Adams H, Lamb AD, Dunning MJ, Halim S, Lindberg J, Massie CM, et al. Integration of copy number and transcriptomics provides risk stratification in prostate cancer: A discovery and validation cohort study. EBioMedicine. 2015;2(9):1133-44.

16. Chen X, Corbin JM, Tipton GJ, Yang LV, Asch AS, Ruiz-Echevarria MJ. The TMEFF2 tumor suppressor modulates integrin expression, RhoA activation and migration of prostate cancer cells. Biochim Biophys Acta. 2014;1843(6):1216-24.

17. Corbin JM, Overcash RF, Wren JD, Coburn A, Tipton GJ, Ezzell JA, et al. Analysis of TMEFF2 allografts and transgenic mouse models reveals roles in prostate regeneration and cancer. Prostate. 2016;76(1):97-113.

18. Green T, Chen X, Ryan S, Asch AS, Ruiz-Echevarria MJ. TMEFF2 and SARDH cooperate to modulate one-carbon metabolism and invasion of prostate cancer cells. Prostate. 2013;73(14):1561-75.

19. Kim JH, Dhanasekaran SM, Prensner JR, Cao X, Robinson D, Kalyana-Sundaram S, et al. Deep sequencing reveals distinct patterns of DNA methylation in prostate cancer. Genome research. 2011;21(7):1028-41.

20. Fournier Pierrick GJ, Juárez P, Jiang G, Clines Gregory A, Niewolna M, Kim Hun S, et al. The TGF-&#x3b2; Signaling Regulator PMEPA1 Suppresses Prostate Cancer Metastases to Bone. Cancer cell.27(6):809-21.

21. McNair C, Urbanucci A, Comstock CES, Augello MA, Goodwin JF, Launchbury R, et al. Cell-cycle coupled expansion of AR activity promotes cancer progression. Oncogene. 2017;36(12):1655-68.

22. Xu Y, Chen SY, Ross KN, Balk SP. Androgens induce prostate cancer cell proliferation through mammalian target of rapamycin activation and post-transcriptional increases in cyclin D proteins. Cancer research. 2006;66(15):7783-92.

23. Balk SP, Knudsen KE. AR, the cell cycle, and prostate cancer. Nuclear Receptor Signaling. 2008;6:e001.

24. Sivanandam A, Murthy S, Kim SH, Barrack ER, Veer Reddy GP. Role of androgen receptor in prostate cancer cell cycle regulation: interaction with cell cycle regulatory proteins and enzymes of DNA synthesis. Current protein & peptide science. 2010;11(6):451-8.

25. Koryakina Y, Knudsen KE, Gioeli D. Cell-cycle-dependent regulation of androgen receptor function. Endocrine-Related Cancer. 2015;22(2):249-64.

26. You S, Knudsen BS, Erho N, Alshalalfa M, Takhar M, Al-Deen Ashab H, et al. Integrated Classification of Prostate Cancer Reveals a Novel Luminal Subtype with Poor Outcome. Cancer research. 2016;76(17):4948-58.

27. Beltran H, Rickman DS, Park K, Chae SS, Sboner A, MacDonald TY, et al. Molecular characterization of neuroendocrine prostate cancer and identification of new drug targets. Cancer Discov. 2011;1(6):487-95.

**SUPPLEMENTARY FIGURES**

A
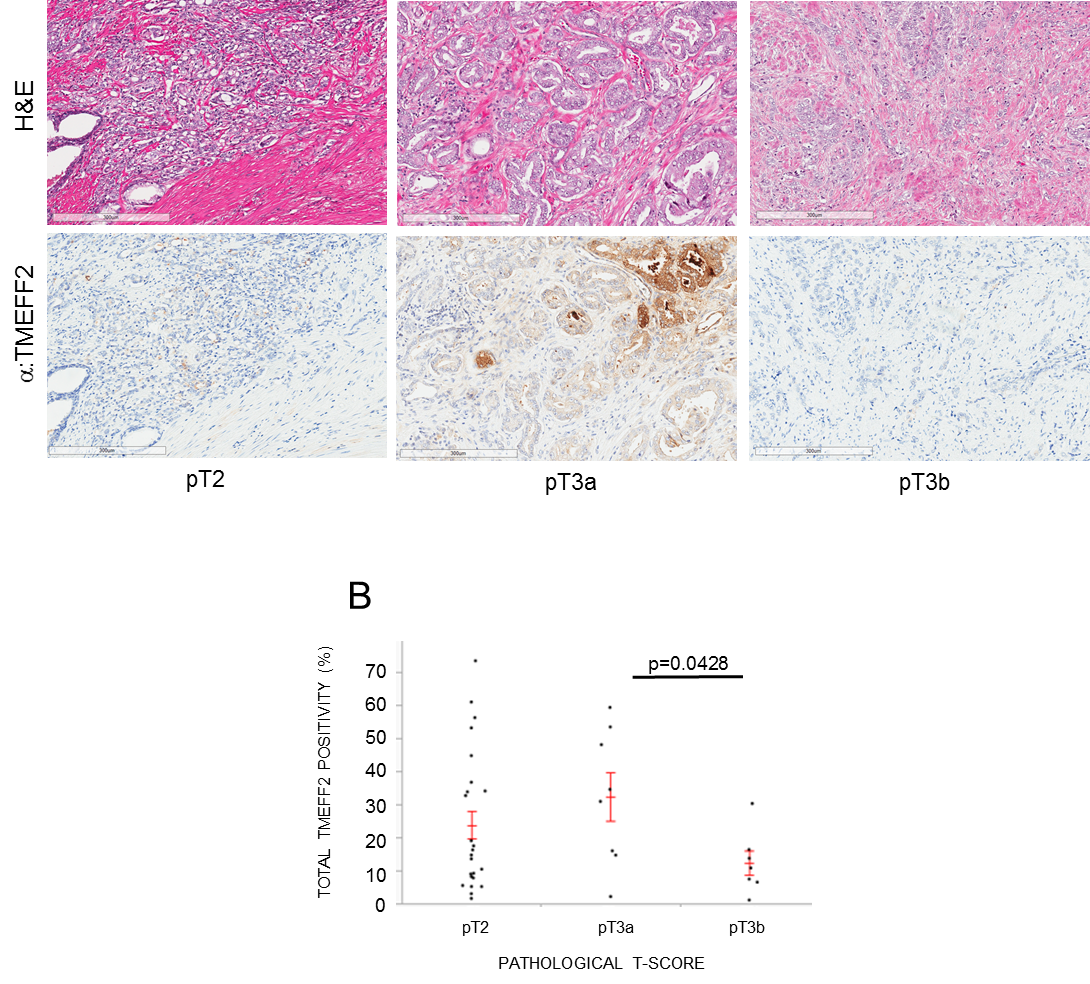


**Figure S1. Expression of TMEFF2 protein in prostate cancer S1A)** H&E and TMEFF2 immunohistochemistry in patient samples from Oklahoma University (OU). Within prostate specimens, TMEFF2 expression is focal, restricted to epithelial luminal cells and its cellular distribution indicates that it is mainly membranous and/or cytoplasmic. Representative examples of samples with distinct Gleason patterns and pathological scores were selected. **S1B)** Quantification of TMEFF2 staining in OU samples of different pathological T score. Statistical analysis was done using a Wilcoxon multiple comparison test.

**
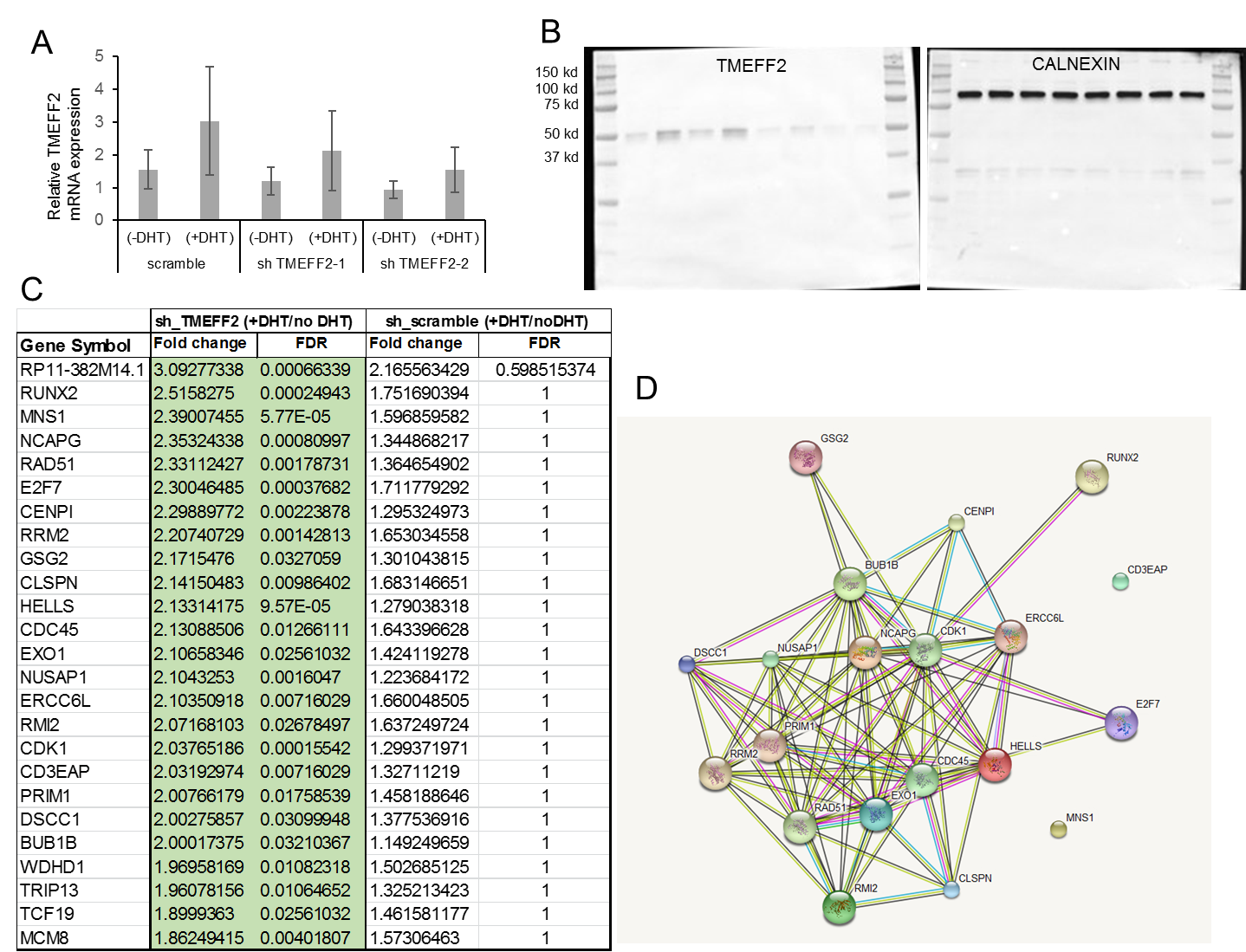
**

**Figure S2. Androgen-induction of nuclear genes is affected by TMEFF2 silencing. S2A)** *TMEFF2* mRNA levels in LNCaP cells transduced with the TMEFF2 silencing shRNAs or the scramble control and grown in the presence or absence of DHT. Note that *Tmeff2* is an androgen regulated gene. **S2B)** Western Blot analysis to determine knockdown of TMEFF2 in LNCaP cells using three different *TMEFF2* targeted shRNAs. Full blots are shown. **S2C)** RNA-seq data showing increased androgen induction of cell cycle related genes in LNCaP cells in which *TMEFF2* has been silenced thru shRNA (sh_TMEFF2) when compared to the cells expressing sh_scramble control. **S2D)** Molecular network analysis of the genes identified as differentially modulated by androgens in *TMEFF2* knock down cells (using the STRING database).


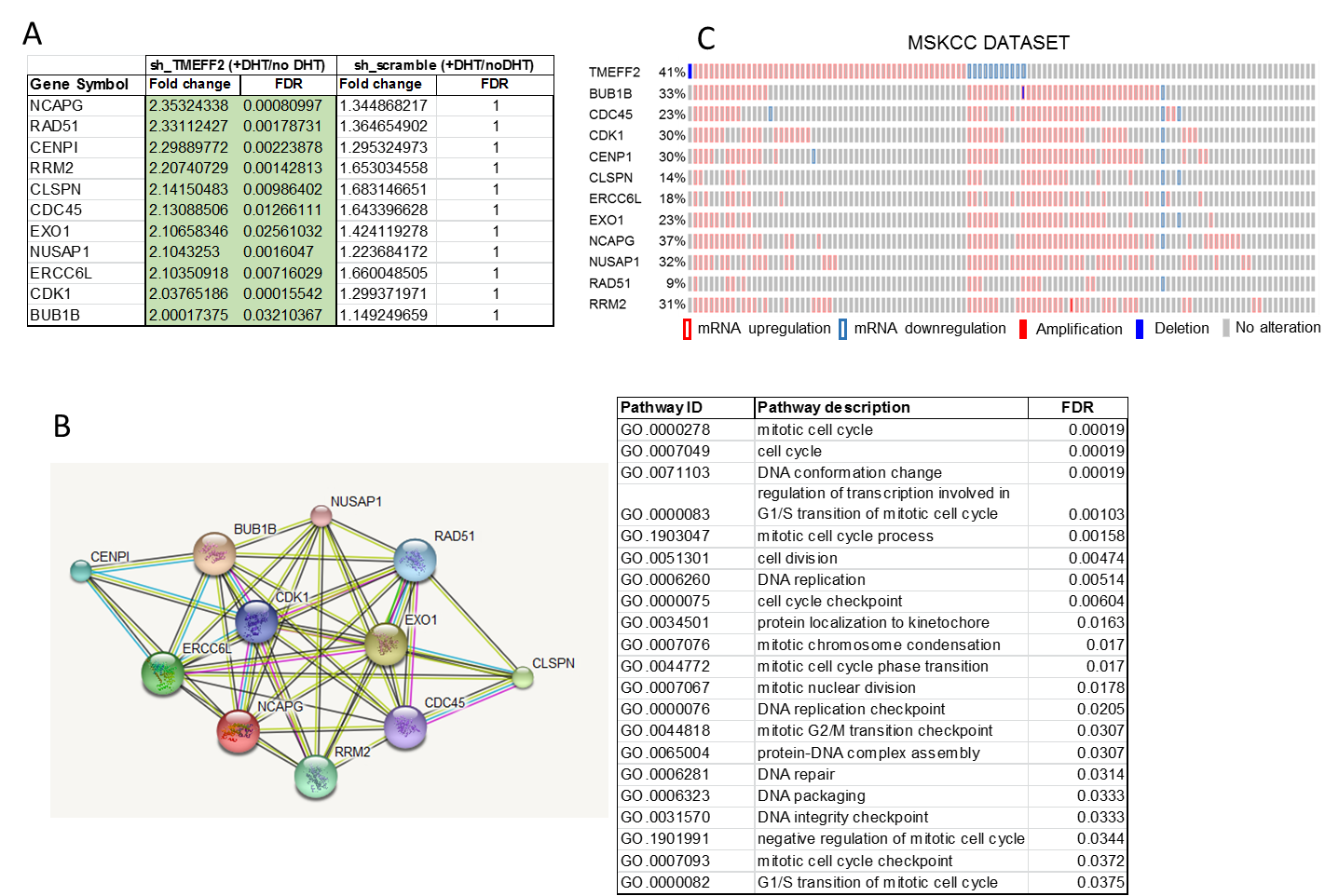


**Figure S3. Selection of the TMEFF2 modulated cell cycle (TMCC11) gene subset. S3A)** the table shows the 11 genes selected as the TMCC11 signature and the fold change in response to DHT treatment in LNCaP expressing the sh_TMEFF2 RNA or the scramble control. **S3B)** Network and enrichment analysis of the TMCC11 signature genes (using the STRING database). **S3C)** Oncoprint of the TMCC11 signature. Data for *TMEFF2* and the 11 mRNAs included in the TMCC11 signature were extracted from MSKCC Prostate project through cBioPortal using a Z-score threshold of ± 1.6 as compared with normal prostate samples, and presented as an OncoPrint (for clarity, most cases without alterations have not been included in the graph). Only tumor samples with mRNA data were selected (n=150). The samples are displayed in columns and arranged to emphasize common changes among the TMCC11 genes, as well as the inverse correlation with low *TMEFF2* expression samples. The alteration percentage for each gene is included left of the oncoprint. Of note, most changes observed in all the genes correspond to mRNA upregulation.

**
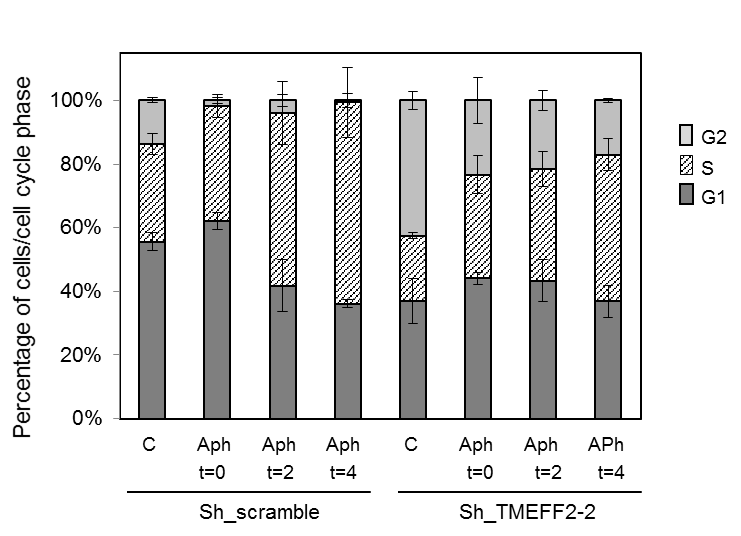
**

**Figure S4. Effect of TMEFF2 silencing on cell cycle progression.** Cell cycle analysis of 22Rv1 cells transduced with scramble control or TMEFF2-2 silencing shRNAs. The percentage of cells in each phase of the cycle under the specific treatments is indicated in the Y- axis. C=control (DMSO), Aph=cells treated with aphidicolin (2 μg/ml), and released from treatment for the indicated amount of time (t; in hours).

**
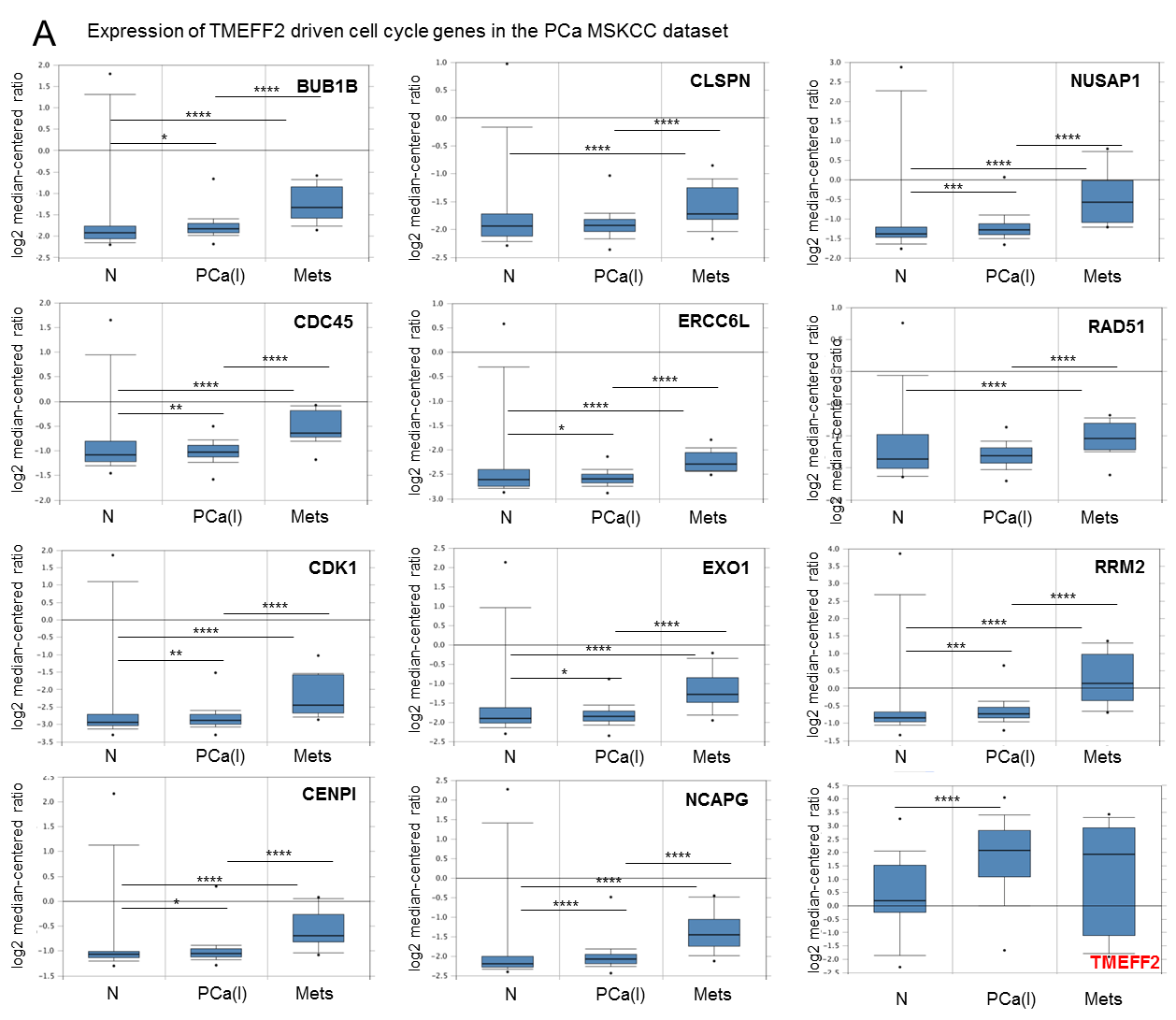
**

**Figure S5A. The TMCC11 signature genes are highly expressed in metastatic prostate cancer.** Expression levels of the individual TMCC11 genes in benign, localized and metastatic prostate samples from patients from the PCa MSKCC dataset. N: normal, PCa(l): localized PCa, Mets: Metastatic PCa. Expression levels are presented as boxplots and statistical analysis was done using a Wilcoxon multiple comparison test. Data obtained from the Oncomine database.

**
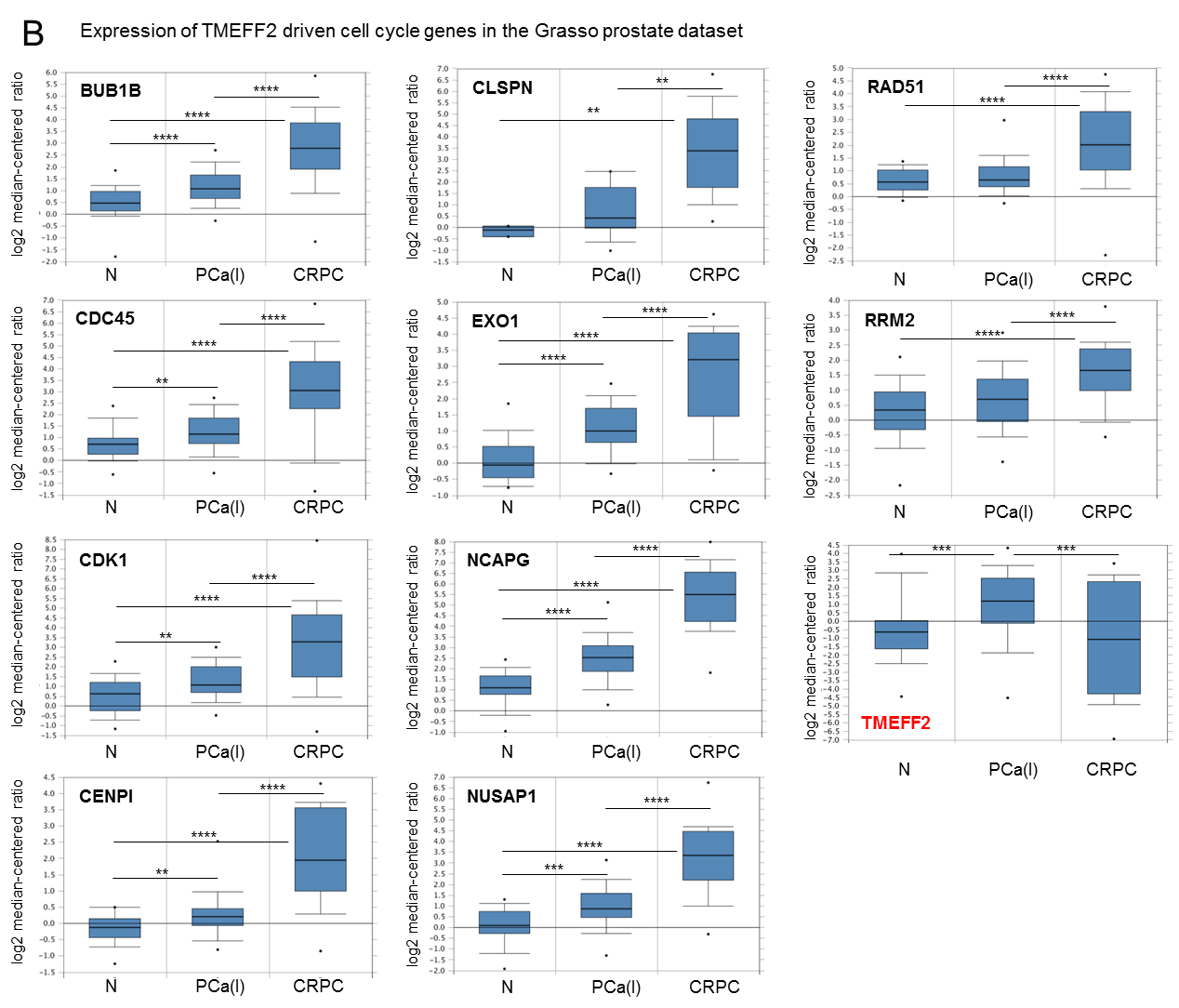
**

**Figure S5B. The TMCC11 signature genes are highly expressed in clinical CRPC.** Expression levels of the individual TMCC11 genes in benign, localized and metastatic CRPC prostate samples from patients from the PCa Grasso dataset (note that no probe is present for the ERCC6L in this dataset). N: normal, PCa(l): localized PCa, CRPC: Castration resistant PCa. Expression levels are presented as boxplots and statistical analysis was done using a Wilcoxon multiple comparison test. Data obtained from the Oncomine database.

**
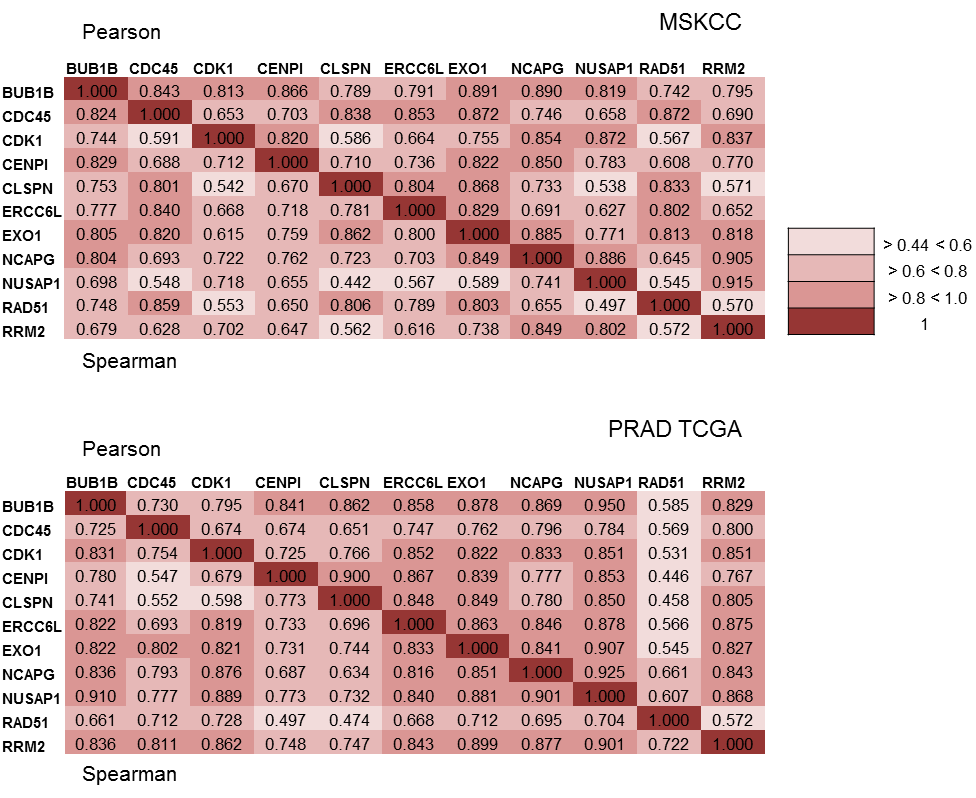
**

**Figure S6. The genes in the TMCC11 signature are significantly co-expressed.** Pearson and Spearman co-expression correlation coefficients are provided for pairwise associations on the levels of the different TMCC11 mRNAs in the PCa MSKCC **(A)** and the PRAD TCGA datasets **(B)**. Association between the expression of the TMCC11 genes was assessed through the cBioportal software using co-expression analysis.

**
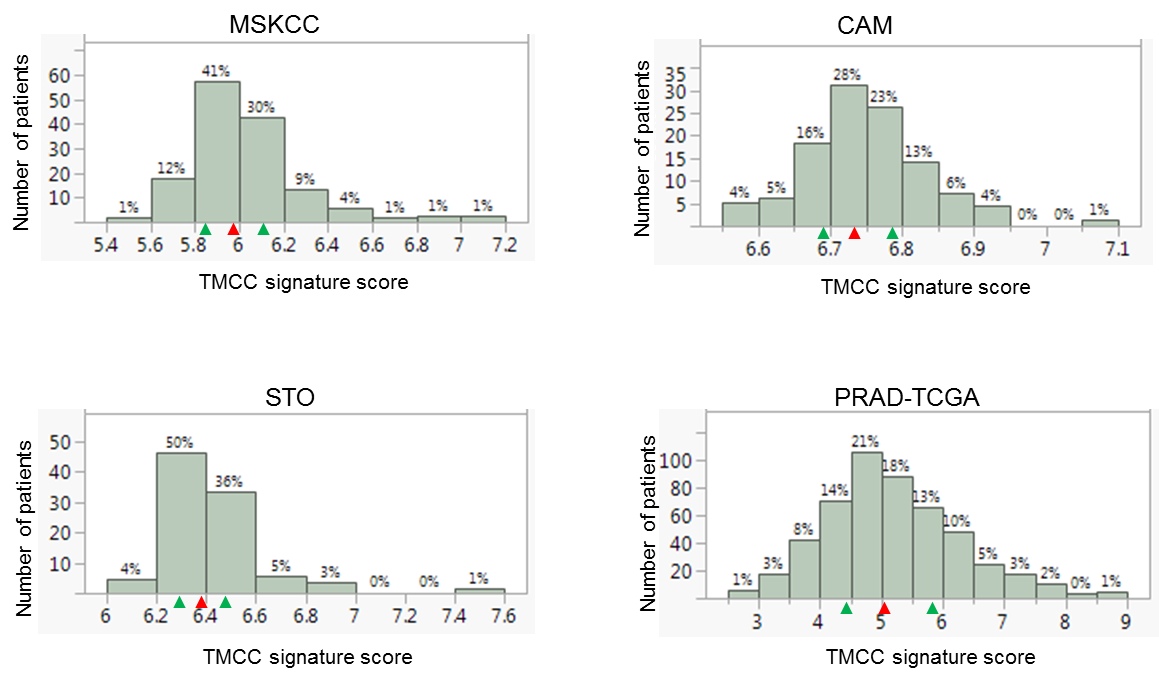
**

**Figure S7. Distribution of the TMCC11 signature score in patients from the different datasets used in this study.** Median (red arrowhead) and quartiles (green arrowheads) are marked.

**
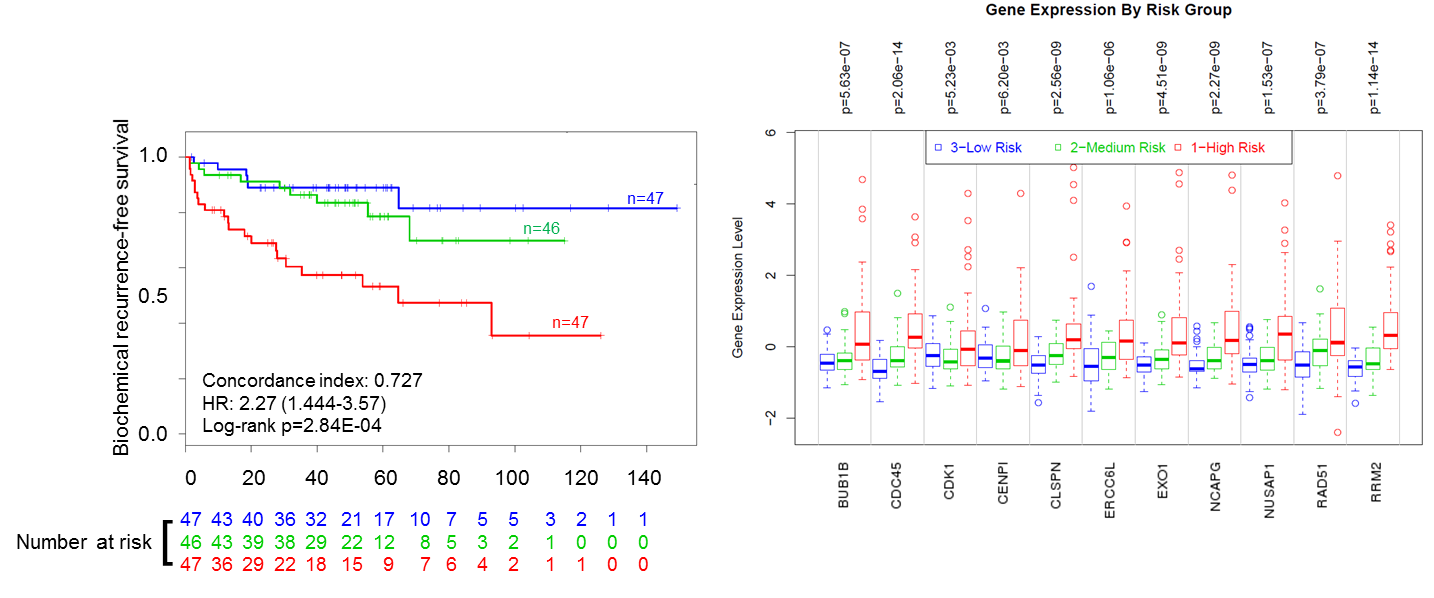
**

**Figure S8. High expression of TMCC11 correlates with poor prognosis in the MSKCC dataset using the SurvExpress platform for analysis. Left)** Kaplan-Meier survival curves of the MSKCC prostate dataset stratified by risk groups (3 groups of the same size) and censored by biochemical recurrence. CI, Concordance Index; HR, Risk Group Hazard Ratio. **Right)** TMCC11 expression levels stratified by risk groups indicates that high risk of BCR correlates with high expression of the TMCC11 genes. Red, high expression; Green, median expression; Blue, low expression.

**
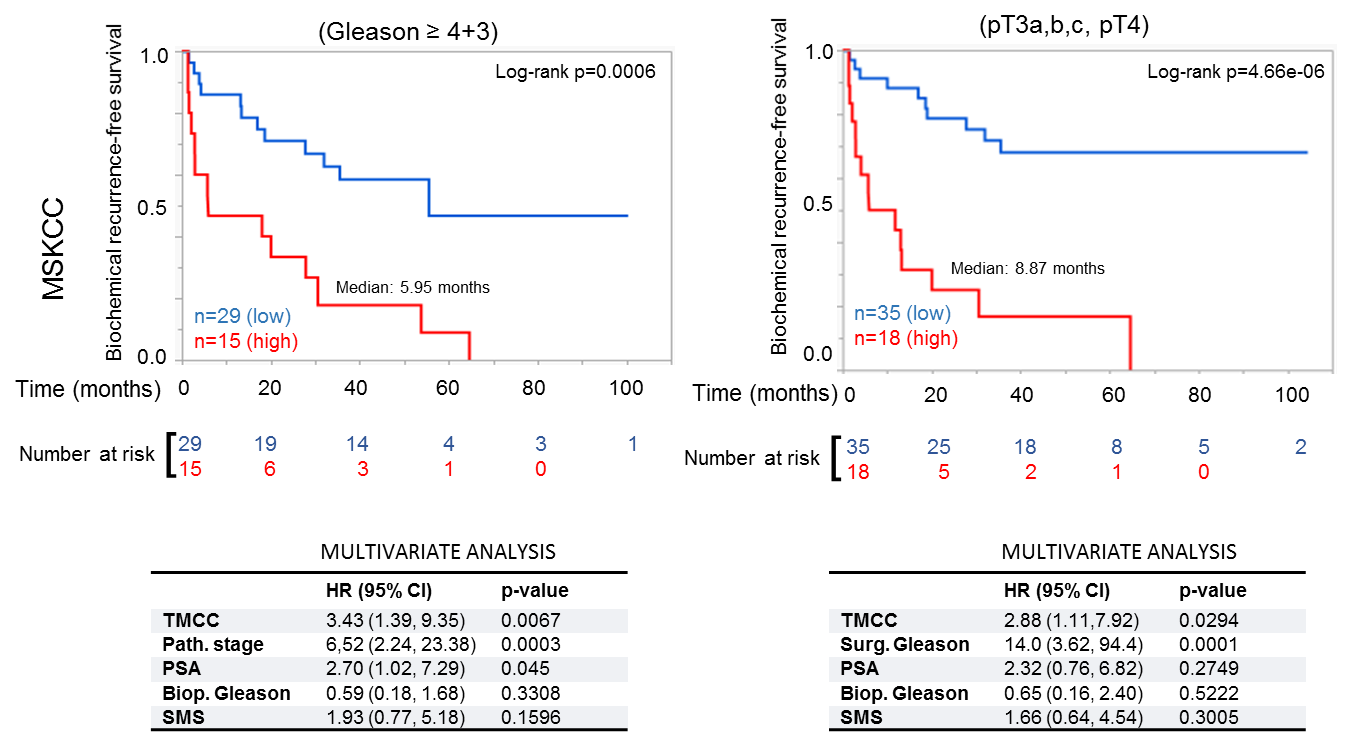
**

**Figure S9. High TMCC11 expression correlates with decreased disease-free survival in subsets of patients with high pathological or surgical Gleason score in the MSKCC dataset.** Kaplan-Meier curves for TMCC11 for BCR endpoint using only samples with high gleason score (≥ 4+3; left panels) or high pathological stage (≥pT3a; right panels). The upper tertile of the TMCC11 was used as the cut point. Red indicates the high TMCC11 group. Tables show multivariate Cox regression analysis of BCR in the same sets of samples. Gleason – High (≥4+3): Low (≤3+4); PSA – High (≥10):Low(<10); Path Stage –High(≥T3):Low(≤T2); Positive surgical margins -Y:N; Extracapsular extension (ECE) – Y:N.

**
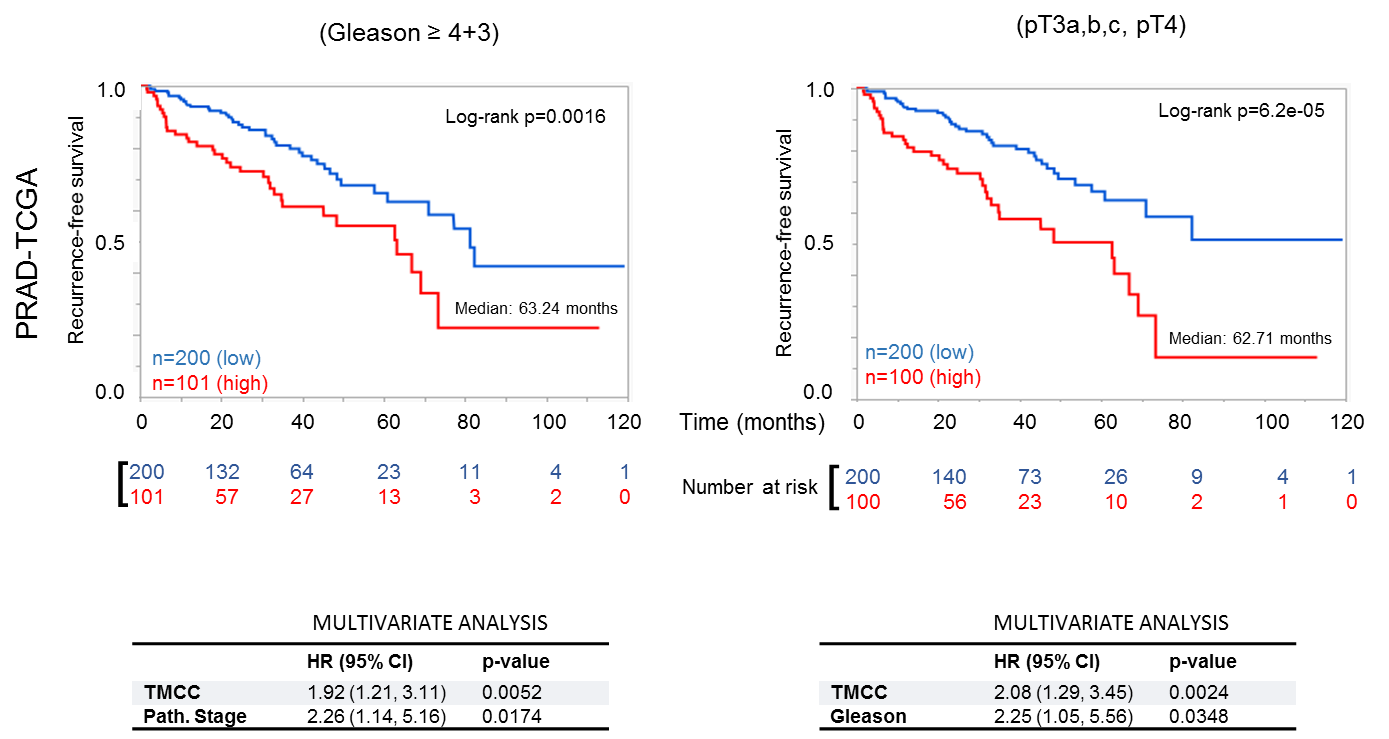
**

**Figure S10. High TMCC11 expression correlates with decreased disease-free survival in subsets of patients with high pathological or surgical Gleason score in the PRAD-TCGA dataset.** Kaplan-Meier curves for TMCC11 for disease recurrence using only samples with high gleason score (≥ 4+3; left panels) or high pathological stage (≥pT3a; right panels). The upper tertile of the TMCC11 was used as the cut point. Red indicates the high TMCC11 group. Tables show multivariate Cox regression analysis of recurrence in the same sets of samples. Gleason – High (≥4+3): Low (≤3+4); PSA – High (≥10):Low(<10); Path Stage –High(≥T3):Low(≤T2); Positive surgical margins -Y:N; Extracapsular extension (ECE) – Y:N.

**
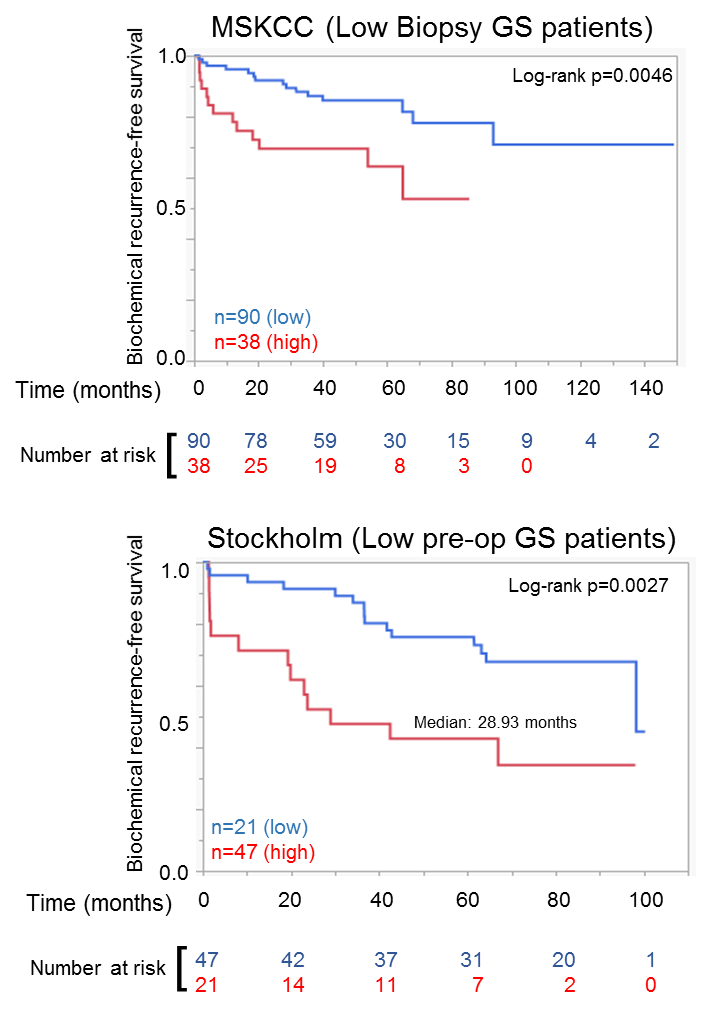
**

**Figure S11. TMCC11 stratifies patients presenting with low biopsy or pre-operative Gleason score.** Kaplan-Meier curves for TMCC11 in the MSKCC (A), and Stockholm (B) datasets, for BCR endpoint using only samples with low biopsy or preoperative gleason score (≤3+4).

**Table S1. List of the 100 most variable expressed genes in 5 different datasets.** Data was obtained from the R2 platform selecting the genes for each dataset that have the highest variation (standard deviation). TMEFF2 is highlighted in yellow.

**Table S2. Overview of clinical datasets used in this study with expression data.**

**
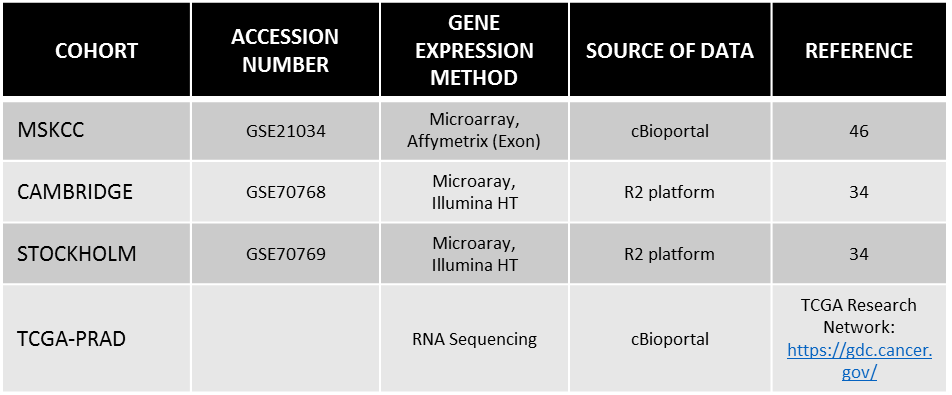
**

**Table S3. Summary of Kaplan-Meier analysis for DFS of the individual 11 genes corresponding to the TMCC11 signature.** The analysis used time to BCR as end point and was conducted in cBioportal using the MSKCC dataset and selecting for patients with expression levels greater than 1.6 standard deviations above the mean as high expressors. Only CLSPN demonstrated a 0.01< p >0.05.

**
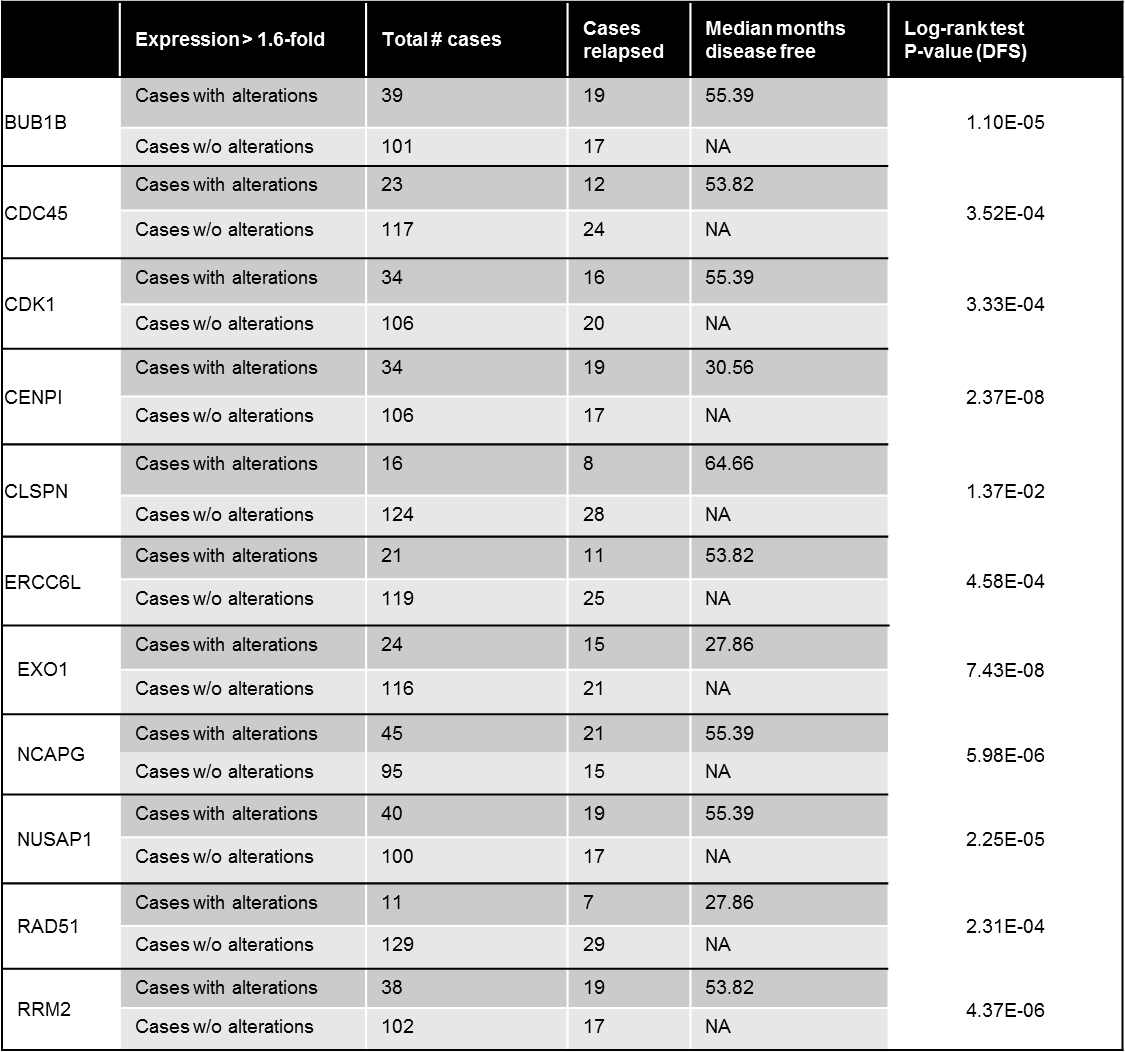
**

**Table S4. C-statistical analysis for time to disease recurrence comparing the performance of TMCC11 alone or in combination with other clinical variables.**


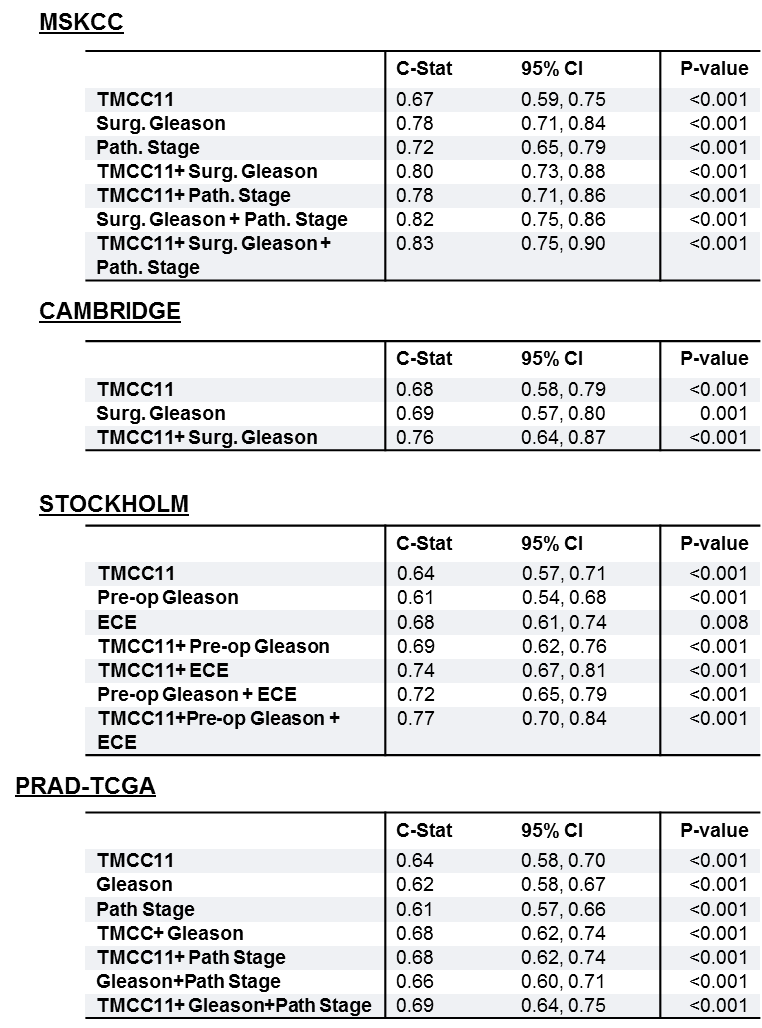


**Table S5. Performance of multiple oncogenic signatures on predicting relapse.** Performance was scored by log-rank test p-value of the difference on time to BCR between high and low risk groups defined by the overall gene expression signature. Data are sorted by first principal component of the individual rankings of the 3 columns corresponding to the Cambridge, Stockholm and MSKCC datasets. The TMCC13 and TMCC3 signatures are derived from TMCC11 (see supplemental methods). Numbers denote p-values.

| **SET** | **pvlGSE70769 (STO)** | **pvlGSE70768 (CAM)** | **pvlGSE21034(MSKCC)** |
| --- | --- | --- | --- |
| _META-PCNA | 0.000322 | 0.0185 | 0.00015 |
| CUZICK | 0.00466 | 0.0161 | 2.10E-06 |
| TMCC13 | 0.00278 | 0.0239 | 3.60E-05 |
| TMCC11 | 0.00915 | 0.00479 | 0.000173 |
| TMCC3 | 0.00627 | 0.0312 | 6.73E-05 |
| _SOTIRIOU-GGI | 0.00128 | 0.0388 | 0.000485 |
| _DAI | 0.00965 | 0.0131 | 0.000199 |
| _MA | 0.0131 | 0.00924 | 0.00265 |
| _CARTER | 0.00378 | 0.00309 | 0.0546 |
| HES6 | 0.00544 | 0.00447 | 0.249 |
| _PEI | 0.0388 | 0.0366 | 0.391 |
| __GCNP_SHH_UP_EARLY.V1_UP | 0.0102 | 0.0126 | 0.518 |
| __CAHOY_NEURONAL | 0.0437 | 0.0339 | 0.508 |
| __MYC_UP.V1_UP | 0.0429 | 0.037 | 0.524 |
| __BCAT.100_UP.V1_DN | 0.0634 | 0.0528 | 0.00929 |
| ross100E | 0.17 | 0.0072 | 0.0607 |
| _TAVAZOIE | 0.0249 | 0.0833 | 0.171 |
| _ABBA | 0.0696 | 0.049 | 0.146 |
| __RB_DN.V1_UP | 0.114 | 0.0255 | 0.165 |
| IRSHAD | 0.141 | 0.0404 | 0.145 |
| __GLI1_UP.V1_UP | 0.0086 | 0.339 | 0.145 |
| __E2F3_UP.V1_UP | 0.0115 | 0.271 | 0.185 |
| __CSR_EARLY_UP.V1_UP | 0.332 | 0.0356 | 0.0906 |
| __EGFR_UP.V1_UP | 0.405 | 0.0627 | 0.0315 |
| __PTEN_DN.V2_UP | 0.0226 | 0.457 | 0.114 |
| __PIGF_UP.V1_UP | 0.244 | 0.0491 | 0.157 |
| _BUFFA | 0.0635 | 0.012 | 0.327 |
| __HINATA_NFKB_IMMU_INF | 0.455 | 0.191 | 0.00702 |
| __ESC_V6.5_UP_LATE.V1_DN | 0.154 | 0.554 | 0.0459 |
| __ATF2_S_UP.V1_DN | 0.0259 | 0.699 | 0.0933 |
| __AKT_UP_MTOR_DN.V1_UP | 0.00628 | 0.216 | 0.435 |
| __CSR_LATE_UP.V1_DN | 0.0296 | 0.443 | 0.236 |
| __LEF1_UP.V1_DN | 0.00708 | 0.44 | 0.307 |
| __RB_P130_DN.V1_DN | 0.0219 | 0.38 | 0.34 |
| _RHODES | 0.0147 | 0.154 | 0.511 |
| __CSR_LATE_UP.V1_UP | 0.00594 | 0.284 | 0.486 |
| __RB_P107_DN.V1_UP | 0.0165 | 0.281 | 0.459 |
| __EIF4E_UP | 0.239 | 0.00106 | 0.437 |
| __ESC_J1_UP_EARLY.V1_DN | 0.0823 | 0.798 | 0.0127 |
| _WONG-ESC | 3.87E-05 | 0.0997 | 0.943 |
| _IVSHINA | 0.00139 | 0.361 | 0.529 |
| _MILLER | 0.411 | 0.509 | 0.0436 |
| __RAF_UP.V1_DN | 0.0428 | 0.593 | 0.229 |
| _CRAWFORD | 0.101 | 0.0489 | 0.769 |
| _PAIK | 0.014 | 0.196 | 0.89 |
| __STK33_NOMO_DN | 0.00622 | 0.71 | 0.328 |
| _GLINSKY | 0.325 | 0.00692 | 0.591 |
| __DCA_UP.V1_DN | 0.0354 | 0.434 | 0.744 |
| __CAMP_UP.V1_DN | 0.865 | 0.483 | 0.0332 |
| __MYC_UP.V1_DN | 0.416 | 0.797 | 0.0488 |
| __SIRNA_EIF4GI_UP | 0.0067 | 0.855 | 0.344 |
| __IL21_UP.V1_UP | 0.0111 | 0.504 | 0.886 |
| __ESC_J1_UP_LATE.V1_DN | 0.795 | 0.627 | 0.018 |
| __CRX_DN.V1_UP | 0.029 | 0.952 | 0.25 |
| rossG1 | 0.0488 | 0.811 | 0.374 |
| __RELA_DN.V1_UP | 0.787 | 0.729 | 0.000785 |
| __E2F1_UP.V1_UP | 0.745 | 0.00813 | 0.513 |
| __PDGF_UP.V1_UP | 0.718 | 0.766 | 0.015 |
| __PKCA_DN.V1_DN | 0.0127 | 0.84 | 0.451 |
| __HOXA9_DN.V1_UP | 0.651 | 0.822 | 0.0367 |
| _RAMASWAMY | 0.925 | 0.739 | 0.035 |
| __WNT_UP.V1_UP | 2.88E-05 | 0.835 | 0.769 |
| __STK33_DN | 0.0461 | 0.816 | 0.724 |
| __JAK2_DN.V1_UP | 0.0296 | 0.859 | 0.755 |
| __PRC1_BMI_UP.V1_UP | 0.0401 | 0.887 | 0.789 |
| _HUA | 0.959 | 0.992 | 0.0171 |
| __P53_DN.V2_DN | 0.0603 | 0.232 | 0.104 |
| ONCOTYPEDX | 0.0538 | 0.156 | 0.206 |
| __PIGF_UP.V1_DN | 0.139 | 0.186 | 0.165 |
| __PKCA_DN.V1_UP | 0.203 | 0.0705 | 0.22 |
| __CRX_NRL_DN.V1_UP | 0.0511 | 0.593 | 0.0707 |
| _VALASTYAN | 0.0926 | 0.203 | 0.241 |
| __TBK1.DF_UP | 0.0502 | 0.366 | 0.222 |
| __E2F1_UP.V1_DN | 0.112 | 0.52 | 0.103 |
| __BRCA1_DN.V1_DN | 0.323 | 0.293 | 0.131 |
| __LTE2_UP.V1_UP | 0.0757 | 0.11 | 0.383 |
| __YAP1_UP | 0.293 | 0.243 | 0.174 |
| __KRAS.300_UP.V1_DN | 0.0645 | 0.19 | 0.367 |
| _SAAL | 0.0515 | 0.268 | 0.361 |
| _BEN-PORATH-EXP1 | 0.0529 | 0.0534 | 0.503 |
| __NOTCH_DN.V1_UP | 0.0522 | 0.315 | 0.362 |
| __TBK1.DN.48HRS_UP | 0.0581 | 0.369 | 0.3 |
| __CAHOY_OLIGODENDROCUTIC | 0.351 | 0.225 | 0.201 |
| __ESC_J1_UP_EARLY.V1_UP | 0.072 | 0.539 | 0.205 |
| __GCNP_SHH_UP_EARLY.V1_DN | 0.239 | 0.435 | 0.179 |
| _KORKOLA | 0.566 | 0.133 | 0.157 |
| __TBK1.DF_DN | 0.445 | 0.22 | 0.175 |
| __RAF_UP.V1_UP | 0.836 | 0.082 | 0.0875 |
| __CRX_NRL_DN.V1_DN | 0.107 | 0.487 | 0.214 |
| __BCAT_BILD_ET_AL_UP | 0.116 | 0.393 | 0.272 |
| __ATF2_UP.V1_DN | 0.22 | 0.612 | 0.115 |
| __BMI1_DN.V1_DN | 0.179 | 0.459 | 0.22 |
| __ERB2_UP.V1_DN | 0.951 | 0.0584 | 0.0653 |
| __HINATA_NFKB_MATRIX | 0.231 | 0.633 | 0.135 |
| __PDGF_ERK_DN.V1_UP | 0.0515 | 0.123 | 0.791 |
| __BCAT_BILD_ET_AL_DN | 0.216 | 0.409 | 0.264 |
| _WHITFIELD | 0.372 | 0.156 | 0.371 |
| __KRAS.BREAST_UP.V1_DN | 0.154 | 0.418 | 0.367 |
| __VEGF_A_UP.V1_UP | 0.0882 | 0.401 | 0.424 |
| __AKT_UP.V1_UP | 0.0583 | 0.302 | 0.58 |
| __NRL_DN.V1_UP | 0.061 | 0.98 | 0.0603 |
| __CORDENONSI_YAP_CONSERVED_SIGNATURE | 0.182 | 0.621 | 0.192 |
| __LEF1_UP.V1_UP | 0.562 | 0.431 | 0.104 |
| __KRAS.DF.V1_UP | 0.606 | 0.125 | 0.23 |
| _KOK | 0.382 | 0.483 | 0.185 |
| __MTOR_UP.V1_DN | 0.0876 | 0.78 | 0.18 |
| __CTIP_DN.V1_DN | 0.229 | 0.703 | 0.166 |
| __IL15_UP.V1_UP | 0.18 | 0.652 | 0.194 |
| __PRC1_BMI_UP.V1_DN | 0.247 | 0.561 | 0.211 |
| rossG2 | 0.0778 | 0.972 | 0.0898 |
| __CYCLIN_D1_UP.V1_DN | 0.389 | 0.314 | 0.326 |
| __MTOR_UP.N4.V1_UP | 0.485 | 0.637 | 0.0524 |
| __VEGF_A_UP.V1_DN | 0.186 | 0.535 | 0.297 |
| __MTOR_UP.N4.V1_DN | 0.673 | 0.118 | 0.261 |
| __IL15_UP.V1_DN | 0.198 | 0.278 | 0.559 |
| __STK33_SKM_UP | 0.264 | 0.81 | 0.103 |
| __GLI1_UP.V1_DN | 0.398 | 0.39 | 0.283 |
| __AKT_UP.V1_DN | 0.167 | 0.89 | 0.106 |
| ross100Both | 0.541 | 0.518 | 0.144 |
| __STK33_NOMO_UP | 0.475 | 0.709 | 0.0642 |
| __CSR_EARLY_UP.V1_DN | 0.95 | 0.148 | 0.184 |
| __LTE2_UP.V1_DN | 0.763 | 0.315 | 0.176 |
| __MEK_UP.V1_DN | 0.456 | 0.336 | 0.29 |
| __AKT_UP_MTOR_DN.V1_DN | 0.823 | 0.209 | 0.215 |
| rossG5 | 0.216 | 0.753 | 0.21 |
| __KRAS.AMP.LUNG_UP.V1_UP | 0.198 | 0.457 | 0.439 |
| __GCNP_SHH_UP_LATE.V1_DN | 0.114 | 0.874 | 0.185 |
| __CYCLIN_D1_KE_.V1_UP | 0.427 | 0.558 | 0.21 |
| __EIF4E_DN | 0.243 | 0.978 | 0.065 |
| _YU | 0.294 | 0.0998 | 0.868 |
| __MEL18_DN.V1_UP | 0.423 | 0.516 | 0.238 |
| rossG4 | 0.0651 | 0.778 | 0.343 |
| __ATM_DN.V1_DN | 0.166 | 0.448 | 0.542 |
| __CAHOY_ASTROCYTIC | 0.239 | 0.527 | 0.397 |
| __KRAS.PROSTATE_UP.V1_DN | 0.369 | 0.234 | 0.559 |
| __KRAS.600.LUNG.BREAST_UP.V1_UP | 0.51 | 0.22 | 0.422 |
| __IL2_UP.V1_UP | 0.418 | 0.731 | 0.163 |
| _SHIPITSIN | 0.594 | 0.48 | 0.206 |
| __PDGF_ERK_DN.V1_DN | 0.842 | 0.574 | 0.0805 |
| __KRAS.AMP.LUNG_UP.V1_DN | 0.302 | 0.47 | 0.422 |
| __ALK_DN.V1_DN | 0.0998 | 0.402 | 0.79 |
| __SIRNA_EIF4GI_DN | 0.308 | 0.861 | 0.14 |
| _WONG-MITOCHON | 0.237 | 0.189 | 0.978 |
| _LIU | 0.696 | 0.531 | 0.16 |
| __ERB2_UP.V1_UP | 0.55 | 0.798 | 0.0506 |
| __BCAT_GDS748_UP | 0.125 | 0.718 | 0.397 |
| __SNF5_DN.V1_UP | 0.382 | 0.587 | 0.284 |
| __PTEN_DN.V2_DN | 0.4 | 0.208 | 0.642 |
| __BMI1_DN_MEL18_DN.V1_DN | 0.381 | 0.359 | 0.508 |
| _PAWITAN | 0.519 | 0.342 | 0.383 |
| __TGFB_UP.V1_DN | 0.452 | 0.284 | 0.5 |
| __TGFB_UP.V1_UP | 0.559 | 0.762 | 0.098 |
| __STK33_UP | 0.521 | 0.801 | 0.0821 |
| _WANG-ALK5T204D | 0.169 | 0.779 | 0.324 |
| __WNT_UP.V1_DN | 0.348 | 0.741 | 0.23 |
| __RELA_DN.V1_DN | 0.0902 | 0.51 | 0.756 |
| __CYCLIN_D1_KE_.V1_DN | 0.681 | 0.112 | 0.474 |
| _WONG-PROTEAS | 0.334 | 0.155 | 0.959 |
| __MEK_UP.V1_UP | 0.896 | 0.577 | 0.0962 |
| __SINGH_KRAS_DEPENDENCY_SIGNATURE_ | 0.162 | 0.385 | 0.936 |
| __CRX_DN.V1_DN | 0.364 | 0.944 | 0.136 |
| __KRAS.LUNG_UP.V1_UP | 0.308 | 0.498 | 0.489 |
| _ADORNO | 0.559 | 0.516 | 0.254 |
| __KRAS.LUNG_UP.V1_DN | 0.506 | 0.295 | 0.502 |
| _WANG-76 | 0.394 | 0.355 | 0.578 |
| __NRL_DN.V1_DN | 0.393 | 0.986 | 0.0934 |
| _CHANG | 0.459 | 0.852 | 0.137 |
| __RB_DN.V1_DN | 0.942 | 0.578 | 0.122 |
| __BCAT_GDS748_DN | 0.768 | 0.336 | 0.349 |
| _BEN-PORATH-PRC2 | 0.604 | 0.385 | 0.404 |
| SHARMA | 0.466 | 0.297 | 0.604 |
| __BRCA1_DN.V1_UP | 0.997 | 0.312 | 0.247 |
| _CHI | 0.847 | 0.549 | 0.191 |
| __ESC_J1_UP_LATE.V1_UP | 0.414 | 0.815 | 0.199 |
| __RPS14_DN.V1_DN | 0.558 | 0.268 | 0.577 |
| __RB_P107_DN.V1_DN | 0.644 | 0.153 | 0.632 |
| _REUTER | 0.448 | 0.794 | 0.21 |
| __BMI1_DN.V1_UP | 0.583 | 0.757 | 0.183 |
| __IL21_UP.V1_DN | 0.411 | 0.33 | 0.761 |
| __RB_P130_DN.V1_UP | 0.216 | 0.779 | 0.405 |
| __STK33_SKM_DN | 0.519 | 0.579 | 0.34 |
| __P53_DN.V2_UP | 0.995 | 0.167 | 0.391 |
| __JAK2_DN.V1_DN | 0.897 | 0.468 | 0.234 |
| __PRC2_EZH2_UP.V1_DN | 0.512 | 0.924 | 0.135 |
| _SOTIRIOU-93 | 0.604 | 0.629 | 0.246 |
| __BMI1_DN_MEL18_DN.V1_UP | 0.683 | 0.913 | 0.0596 |
| rossG3 | 0.973 | 0.485 | 0.205 |
| __ESC_V6.5_UP_EARLY.V1_DN | 0.903 | 0.76 | 0.0925 |
| _WELM | 0.295 | 0.467 | 0.861 |
| __PRC2_SUZ12_UP.V1_DN | 0.934 | 0.323 | 0.369 |
| _TAUBE | 0.752 | 0.824 | 0.107 |
| __GCNP_SHH_UP_LATE.V1_UP | 0.8 | 0.212 | 0.568 |
| _SORLIE | 0.547 | 0.952 | 0.154 |
| __BCAT.100_UP.V1_UP | 0.163 | 0.92 | 0.422 |
| __PRC2_EED_UP.V1_UP | 0.965 | 0.632 | 0.172 |
| __CAHOY_ASTROGLIAL | 0.164 | 0.895 | 0.429 |
| __KRAS.600.LUNG.BREAST_UP.V1_DN | 0.354 | 0.441 | 0.917 |
| _BUESS | 0.413 | 0.341 | 0.994 |
| __E2F3_UP.V1_DN | 0.293 | 0.603 | 0.743 |
| __SRC_UP.V1_UP | 0.73 | 0.714 | 0.222 |
| __IL2_UP.V1_DN | 0.162 | 0.645 | 0.912 |
| __KRAS.50_UP.V1_DN | 0.501 | 0.437 | 0.692 |
| __KRAS.LUNG.BREAST_UP.V1_UP | 0.899 | 0.0766 | 0.781 |
| __KRAS.600_UP.V1_DN | 0.437 | 0.429 | 0.91 |
| __ATF2_UP.V1_UP | 0.857 | 0.704 | 0.228 |
| __NFE2L2.V2 | 0.411 | 0.882 | 0.306 |
| __KRAS.KIDNEY_UP.V1_UP | 0.318 | 0.625 | 0.763 |
| LALONDE | 0.687 | 0.736 | 0.282 |
| __TBK1.DN.48HRS_DN | 0.266 | 0.781 | 0.565 |
| __CAMP_UP.V1_UP | 0.74 | 0.387 | 0.583 |
| __ESC_V6.5_UP_LATE.V1_UP | 0.252 | 0.97 | 0.414 |
| _VANTVEER | 0.129 | 0.804 | 0.85 |
| __MEL18_DN.V1_DN | 0.462 | 0.956 | 0.247 |
| _HALLSTROM | 0.346 | 0.575 | 0.996 |
| __P53_DN.V1_UP | 0.626 | 0.564 | 0.515 |
| __HOXA9_DN.V1_DN | 0.297 | 0.782 | 0.693 |
| _HE | 0.581 | 0.746 | 0.408 |
| __PRC2_EZH2_UP.V1_UP | 0.611 | 0.401 | 0.897 |
| __PDGF_UP.V1_DN | 0.648 | 0.724 | 0.401 |
| __CYCLIN_D1_UP.V1_UP | 0.526 | 0.611 | 0.624 |
| __KRAS.DF.V1_DN | 0.9 | 0.843 | 0.185 |
| __KRAS.BREAST_UP.V1_UP | 0.436 | 0.539 | 0.995 |
| __ALK_DN.V1_UP | 0.858 | 0.946 | 0.173 |
| _HU | 0.306 | 0.866 | 0.561 |
| __ATM_DN.V1_UP | 0.852 | 0.81 | 0.245 |
| __SNF5_DN.V1_DN | 0.395 | 0.961 | 0.425 |
| __EGFR_UP.V1_DN | 0.954 | 0.667 | 0.343 |
| __CTIP_DN.V1_UP | 0.857 | 0.587 | 0.481 |
| __NOTCH_DN.V1_DN | 0.471 | 0.836 | 0.461 |
| _MORI | 0.864 | 0.638 | 0.432 |
| __P53_DN.V1_DN | 0.623 | 0.85 | 0.387 |
| __SRC_UP.V1_DN | 0.693 | 0.985 | 0.241 |
| __PRC2_EED_UP.V1_DN | 0.749 | 0.945 | 0.28 |
| __PRC2_SUZ12_UP.V1_UP | 0.61 | 0.93 | 0.374 |
| __YAP1_DN | 0.402 | 0.925 | 0.629 |
| _WEST | 0.935 | 0.785 | 0.384 |
| __PTEN_DN.V1_UP | 0.883 | 0.978 | 0.26 |
| __RAPA_EARLY_UP.V1_DN | 0.99 | 0.679 | 0.503 |
| __ESC_V6.5_UP_EARLY.V1_UP | 0.943 | 0.766 | 0.437 |
| __KRAS.KIDNEY_UP.V1_DN | 0.789 | 0.595 | 0.876 |
| __RAPA_EARLY_UP.V1_UP | 0.812 | 0.723 | 0.673 |
| __JNK_DN.V1_DN | 0.667 | 0.741 | 0.779 |
| __KRAS.LUNG.BREAST_UP.V1_DN | 0.66 | 0.794 | 0.646 |
| __KRAS.50_UP.V1_UP | 0.725 | 0.62 | 0.971 |
| __RPS14_DN.V1_UP | 0.972 | 0.753 | 0.476 |
| __KRAS.600_UP.V1_UP | 0.86 | 0.811 | 0.467 |
| __KRAS.PROSTATE_UP.V1_UP | 0.715 | 0.847 | 0.543 |
| __JNK_DN.V1_UP | 0.637 | 0.741 | 0.928 |
| __DCA_UP.V1_UP | 0.999 | 0.621 | 0.701 |
| __ATF2_S_UP.V1_UP | 0.495 | 0.97 | 0.711 |
| __MTOR_UP.V1_UP | 0.495 | 0.977 | 0.819 |
| __KRAS.300_UP.V1_UP | 0.62 | 0.863 | 0.827 |
| __PTEN_DN.V1_DN | 0.935 | 0.911 | 0.733 |

**Table S6. Comparison of the prognostic potential for relapse of multiple oncogenic signatures against random sets of genes.** For each signature, 10,000 equal size signatures were generated at random and evaluated for predicting early relapse by log-rank test p-value. An overall bootstrap p-value score was computed as proportion of random signatures performing better than the initial signature. Data are sorted by first principal component of the individual rankings of the 3 columns corresponding to the Cambridge, Stockholm and MSKCC datasets. The TMCC13 and TMCC3 signatures are derived from TMCC11 (see supplemental methods). Numbers denote p-values.

| **SET** | **GSE70769 (STO)** | **GSE70768 (CAM)** | **GSE21034 (MSKCC)** |
| --- | --- | --- | --- |
| _META-PCNA | 0.0018 | 0.0355 | 0.00000 |
| CUZICK | 0.0182 | 0.0272 | 0.00000 |
| TMCC13 | 0.0149 | 0.0362 | 0.0005 |
| TMCC11 | 0.0305 | 0.012 | 0.0018 |
| TMCC3 | 0.018 | 0.0375 | 0.0011 |
| _DAI | 0.0314 | 0.0252 | 0.0007 |
| _SOTIRIOU-GGI | 0.0083 | 0.0647 | 0.0001 |
| _CARTER | 0.0143 | 0.0078 | 0.1203 |
| _MA | 0.0366 | 0.0171 | 0.0071 |
| HES6 | 0.0242 | 0.0126 | 0.5834 |
| __GCNP_SHH_UP_EARLY.V1_UP | 0.0303 | 0.0267 | 0.8336 |
| ross100E | 0.2609 | 0.0173 | 0.1388 |
| _BUFFA | 0.1047 | 0.0161 | 0.491 |
| __GLI1_UP.V1_UP | 0.025 | 0.374 | 0.2821 |
| __RB_DN.V1_UP | 0.1916 | 0.0493 | 0.3961 |
| _PEI | 0.0731 | 0.0444 | 0.552 |
| __E2F3_UP.V1_UP | 0.0337 | 0.3192 | 0.4658 |
| _RHODES | 0.0422 | 0.196 | 0.7346 |
| _IVSHINA | 0.0101 | 0.3853 | 0.6659 |
| __AKT_UP_MTOR_DN.V1_UP | 0.0211 | 0.2641 | 0.784 |
| __EIF4E_UP | 0.3347 | 0.0035 | 0.713 |
| __LEF1_UP.V1_DN | 0.0243 | 0.4855 | 0.6621 |
| __RB_P107_DN.V1_UP | 0.0443 | 0.3317 | 0.7679 |
| __CSR_LATE_UP.V1_UP | 0.0213 | 0.3334 | 0.8096 |
| _GLINSKY | 0.4168 | 0.0153 | 0.7177 |
| _WONG-ESC | 6.00E-04 | 0.1444 | 0.9947 |
| _PAIK | 0.0423 | 0.2266 | 0.9244 |
| __STK33_NOMO_DN | 0.0235 | 0.7313 | 0.7445 |
| __SIRNA_EIF4GI_UP | 0.0247 | 0.8694 | 0.6231 |
| __IL21_UP.V1_UP | 0.0327 | 0.5388 | 0.976 |
| __RELA_DN.V1_UP | 0.8236 | 0.7517 | 0.0001 |
| __PKCA_DN.V1_DN | 0.0351 | 0.8532 | 0.7713 |
| __E2F1_UP.V1_UP | 0.7986 | 0.021 | 0.8417 |
| __WNT_UP.V1_UP | 3.00E-04 | 0.8488 | 0.9417 |
| _HUA | 0.9613 | 0.9932 | 0.0032 |
| __BCAT.100_UP.V1_DN | 0.125 | 0.077 | 0.0224 |
| _TAVAZOIE | 0.0625 | 0.1045 | 0.3121 |
| _ABBA | 0.1303 | 0.0761 | 0.3367 |
| IRSHAD | 0.2208 | 0.0552 | 0.2819 |
| __P53_DN.V2_DN | 0.1135 | 0.2786 | 0.2535 |
| ONCOTYPEDX | 0.1126 | 0.1846 | 0.3586 |
| __CSR_EARLY_UP.V1_UP | 0.4284 | 0.0621 | 0.2317 |
| _VALASTYAN | 0.1582 | 0.223 | 0.392 |
| __EGFR_UP.V1_UP | 0.5015 | 0.0969 | 0.0568 |
| __PTEN_DN.V2_UP | 0.0563 | 0.4977 | 0.2726 |
| __PIGF_UP.V1_UP | 0.3443 | 0.0799 | 0.3945 |
| __PIGF_UP.V1_DN | 0.2279 | 0.236 | 0.4173 |
| __HINATA_NFKB_IMMU_INF | 0.5411 | 0.2196 | 0.0229 |
| __PKCA_DN.V1_UP | 0.2934 | 0.1076 | 0.5149 |
| __CRX_NRL_DN.V1_UP | 0.1062 | 0.6241 | 0.1686 |
| __CAHOY_NEURONAL | 0.0942 | 0.0585 | 0.7649 |
| __ESC_V6.5_UP_LATE.V1_DN | 0.2427 | 0.5857 | 0.0937 |
| __ATF2_S_UP.V1_DN | 0.0613 | 0.7166 | 0.2373 |
| __YAP1_UP | 0.3977 | 0.2815 | 0.3472 |
| __E2F1_UP.V1_DN | 0.1907 | 0.5563 | 0.2595 |
| __TBK1.DN.48HRS_UP | 0.1183 | 0.4149 | 0.5175 |
| __BRCA1_DN.V1_DN | 0.4171 | 0.3394 | 0.3111 |
| _SAAL | 0.1034 | 0.3161 | 0.6932 |
| __MYC_UP.V1_UP | 0.0909 | 0.0634 | 0.8383 |
| __KRAS.300_UP.V1_DN | 0.1226 | 0.2392 | 0.6958 |
| __CSR_LATE_UP.V1_DN | 0.0666 | 0.4837 | 0.5388 |
| __RB_P130_DN.V1_DN | 0.0556 | 0.4247 | 0.6628 |
| __LTE2_UP.V1_UP | 0.143 | 0.1569 | 0.7475 |
| __BCAT_BILD_ET_AL_UP | 0.1894 | 0.4385 | 0.4784 |
| __TBK1.DF_UP | 0.107 | 0.4111 | 0.5779 |
| __CAHOY_OLIGODENDROCUTIC | 0.4456 | 0.2696 | 0.4318 |
| __ESC_J1_UP_EARLY.V1_DN | 0.1514 | 0.8122 | 0.0138 |
| _KORKOLA | 0.626 | 0.1535 | 0.2979 |
| __NOTCH_DN.V1_UP | 0.1057 | 0.3637 | 0.7246 |
| _BEN-PORATH-EXP1 | 0.1063 | 0.0852 | 0.8993 |
| __CRX_NRL_DN.V1_DN | 0.1851 | 0.5357 | 0.4731 |
| _MILLER | 0.5022 | 0.5372 | 0.1044 |
| __RAF_UP.V1_UP | 0.8636 | 0.1194 | 0.2272 |
| __BCAT_BILD_ET_AL_DN | 0.3141 | 0.4507 | 0.4672 |
| __ESC_J1_UP_EARLY.V1_UP | 0.1375 | 0.5746 | 0.5013 |
| __GCNP_SHH_UP_EARLY.V1_DN | 0.3291 | 0.4782 | 0.4463 |
| __HINATA_NFKB_MATRIX | 0.3157 | 0.6467 | 0.253 |
| __ATF2_UP.V1_DN | 0.3123 | 0.6348 | 0.2834 |
| __BMI1_DN.V1_DN | 0.264 | 0.5043 | 0.4969 |
| __ERB2_UP.V1_DN | 0.9619 | 0.0927 | 0.1514 |
| __RAF_UP.V1_DN | 0.0927 | 0.6233 | 0.5424 |
| __PDGF_ERK_DN.V1_UP | 0.0993 | 0.1665 | 0.9365 |
| __CORDENONSI_YAP_CONSERVED_SIGNATURE | 0.278 | 0.6516 | 0.3864 |
| __TBK1.DF_DN | 0.5339 | 0.2702 | 0.5019 |
| __GLI1_UP.V1_DN | 0.4928 | 0.4256 | 0.4619 |
| __KRAS.BREAST_UP.V1_DN | 0.2371 | 0.4648 | 0.6927 |
| __AKT_UP.V1_UP | 0.1159 | 0.353 | 0.8668 |
| _CRAWFORD | 0.1708 | 0.0787 | 0.9734 |
| __LEF1_UP.V1_UP | 0.6444 | 0.4701 | 0.2629 |
| __NRL_DN.V1_UP | 0.1191 | 0.981 | 0.1363 |
| _KOK | 0.474 | 0.5229 | 0.4548 |
| rossG2 | 0.1456 | 0.9759 | 0.2008 |
| __VEGF_A_UP.V1_UP | 0.16 | 0.4447 | 0.7792 |
| __CTIP_DN.V1_DN | 0.32 | 0.7274 | 0.3982 |
| __MTOR_UP.V1_DN | 0.16 | 0.7963 | 0.4558 |
| __KRAS.DF.V1_UP | 0.6789 | 0.1704 | 0.5399 |
| __PRC1_BMI_UP.V1_DN | 0.3451 | 0.594 | 0.5071 |
| __MTOR_UP.N4.V1_UP | 0.5773 | 0.6642 | 0.1074 |
| rossG5 | 0.3108 | 0.7716 | 0.3933 |
| __IL15_UP.V1_UP | 0.2782 | 0.6754 | 0.4743 |
| __CYCLIN_D1_UP.V1_DN | 0.4897 | 0.3622 | 0.6885 |
| __VEGF_A_UP.V1_DN | 0.2841 | 0.5679 | 0.6451 |
| __AKT_UP.V1_DN | 0.2579 | 0.8977 | 0.2675 |
| _YU | 0.3861 | 0.1219 | 0.9098 |
| __IL15_UP.V1_DN | 0.2914 | 0.321 | 0.8648 |
| ross100Both | 0.6154 | 0.548 | 0.3278 |
| __STK33_NOMO_UP | 0.5524 | 0.7328 | 0.1583 |
| __CAMP_UP.V1_DN | 0.8921 | 0.5212 | 0.0641 |
| __MTOR_UP.N4.V1_DN | 0.7373 | 0.1656 | 0.5817 |
| __STK33_SKM_UP | 0.3639 | 0.8241 | 0.2752 |
| __MYC_UP.V1_DN | 0.5074 | 0.8144 | 0.1002 |
| _ADORNO | 0.5986 | 0.5134 | 0.4206 |
| __DCA_UP.V1_DN | 0.0788 | 0.4742 | 0.9355 |
| __KRAS.AMP.LUNG_UP.V1_UP | 0.288 | 0.4976 | 0.7526 |
| __CSR_EARLY_UP.V1_DN | 0.9583 | 0.1931 | 0.4393 |
| rossG4 | 0.1279 | 0.7981 | 0.5681 |
| rossG1 | 0.1042 | 0.8262 | 0.5587 |
| __CAHOY_ASTROCYTIC | 0.3342 | 0.5596 | 0.678 |
| __LTE2_UP.V1_DN | 0.8094 | 0.3597 | 0.4569 |
| __BCAT_GDS748_UP | 0.2019 | 0.7333 | 0.6152 |
| __MEK_UP.V1_DN | 0.5521 | 0.3812 | 0.6357 |
| __AKT_UP_MTOR_DN.V1_DN | 0.8602 | 0.2543 | 0.5174 |
| __EIF4E_DN | 0.3384 | 0.9774 | 0.15 |
| _SHIPITSIN | 0.6618 | 0.5248 | 0.4026 |
| SHARMA | 0.5489 | 0.3234 | 0.7237 |
| __ESC_J1_UP_LATE.V1_DN | 0.8378 | 0.6497 | 0.0187 |
| __ATM_DN.V1_DN | 0.2498 | 0.4899 | 0.8291 |
| __MEL18_DN.V1_UP | 0.5101 | 0.5551 | 0.5264 |
| __CRX_DN.V1_UP | 0.0707 | 0.9546 | 0.5354 |
| _WHITFIELD | 0.472 | 0.2073 | 0.8875 |
| __GCNP_SHH_UP_LATE.V1_DN | 0.193 | 0.8867 | 0.4605 |
| __KRAS.PROSTATE_UP.V1_DN | 0.4613 | 0.2813 | 0.8357 |
| _PAWITAN | 0.602 | 0.3855 | 0.5976 |
| __CYCLIN_D1_KE_.V1_UP | 0.5304 | 0.5914 | 0.504 |
| __ALK_DN.V1_DN | 0.1733 | 0.443 | 0.9355 |
| __KRAS.AMP.LUNG_UP.V1_DN | 0.3917 | 0.5178 | 0.7435 |
| __PTEN_DN.V2_DN | 0.4913 | 0.2566 | 0.8752 |
| __SIRNA_EIF4GI_DN | 0.4056 | 0.8703 | 0.3173 |
| __PDGF_ERK_DN.V1_DN | 0.8695 | 0.6195 | 0.1989 |
| __IL2_UP.V1_UP | 0.5227 | 0.7498 | 0.4099 |
| _WONG-MITOCHON | 0.333 | 0.2354 | 0.9958 |
| __ERB2_UP.V1_UP | 0.6305 | 0.8196 | 0.1008 |
| _WANG-76 | 0.4876 | 0.3998 | 0.7839 |
| __BMI1_DN_MEL18_DN.V1_DN | 0.4737 | 0.4055 | 0.8054 |
| __PDGF_UP.V1_UP | 0.7732 | 0.7832 | 0.0211 |
| _LIU | 0.752 | 0.582 | 0.3936 |
| __SNF5_DN.V1_UP | 0.4837 | 0.6181 | 0.613 |
| __TGFB_UP.V1_UP | 0.6399 | 0.7747 | 0.2499 |
| __STK33_UP | 0.5973 | 0.8195 | 0.2266 |
| _WONG-PROTEAS | 0.4349 | 0.1922 | 0.9804 |
| __SINGH_KRAS_DEPENDENCY_SIGNATURE_ | 0.2491 | 0.4143 | 0.9605 |
| __RELA_DN.V1_DN | 0.1614 | 0.5515 | 0.923 |
| __MEK_UP.V1_UP | 0.9179 | 0.6115 | 0.2456 |
| __WNT_UP.V1_DN | 0.4411 | 0.7521 | 0.5318 |
| __KRAS.600.LUNG.BREAST_UP.V1_UP | 0.5872 | 0.2684 | 0.8243 |
| __BCAT_GDS748_DN | 0.8096 | 0.3787 | 0.5592 |
| __TGFB_UP.V1_DN | 0.5434 | 0.3318 | 0.8336 |
| __NRL_DN.V1_DN | 0.4871 | 0.9859 | 0.2297 |
| __KRAS.LUNG_UP.V1_UP | 0.3991 | 0.538 | 0.7864 |
| __HOXA9_DN.V1_UP | 0.7171 | 0.8412 | 0.0706 |
| __CYCLIN_D1_KE_.V1_DN | 0.7413 | 0.1546 | 0.8144 |
| __CRX_DN.V1_DN | 0.4562 | 0.9498 | 0.3246 |
| __KRAS.LUNG_UP.V1_DN | 0.5903 | 0.3437 | 0.8033 |
| _WANG-ALK5T204D | 0.2604 | 0.7996 | 0.6969 |
| _WELM | 0.3699 | 0.4731 | 0.9058 |
| rossG3 | 0.9796 | 0.5128 | 0.3796 |
| _RAMASWAMY | 0.9394 | 0.7527 | 0.088 |
| __RB_DN.V1_DN | 0.9533 | 0.6135 | 0.2866 |
| __BRCA1_DN.V1_UP | 0.9981 | 0.3635 | 0.5225 |
| _CHI | 0.8769 | 0.5868 | 0.4351 |
| __RB_P107_DN.V1_DN | 0.7119 | 0.203 | 0.8715 |
| __RB_P130_DN.V1_UP | 0.3167 | 0.7987 | 0.7173 |
| __BCAT.100_UP.V1_UP | 0.2483 | 0.9254 | 0.6391 |
| _CHANG | 0.5468 | 0.8667 | 0.3956 |
| __RPS14_DN.V1_DN | 0.6409 | 0.3188 | 0.8733 |
| __ESC_J1_UP_LATE.V1_UP | 0.5129 | 0.8266 | 0.4903 |
| __BMI1_DN.V1_UP | 0.6666 | 0.7751 | 0.4377 |
| __P53_DN.V2_UP | 0.9955 | 0.2185 | 0.7238 |
| __IL21_UP.V1_DN | 0.5068 | 0.3699 | 0.9411 |
| __BMI1_DN_MEL18_DN.V1_UP | 0.7463 | 0.918 | 0.1317 |
| __JAK2_DN.V1_DN | 0.9207 | 0.5149 | 0.5186 |
| __CAHOY_ASTROGLIAL | 0.2517 | 0.9048 | 0.7037 |
| __KRAS.50_UP.V1_DN | 0.5844 | 0.4726 | 0.8311 |
| __ESC_V6.5_UP_EARLY.V1_DN | 0.9244 | 0.7826 | 0.2305 |
| __PRC2_EZH2_UP.V1_DN | 0.5972 | 0.9329 | 0.3426 |
| _BUESS | 0.5009 | 0.3698 | 0.9969 |
| _TAUBE | 0.7974 | 0.8435 | 0.2718 |
| _SORLIE | 0.6191 | 0.955 | 0.2897 |
| __TBK1.DN.48HRS_DN | 0.3657 | 0.8013 | 0.7569 |
| _SOTIRIOU-93 | 0.6694 | 0.6579 | 0.6297 |
| __STK33_SKM_DN | 0.593 | 0.6167 | 0.7535 |
| __GCNP_SHH_UP_LATE.V1_UP | 0.8394 | 0.2608 | 0.873 |
| __PRC2_SUZ12_UP.V1_DN | 0.9471 | 0.3715 | 0.7297 |
| __E2F3_UP.V1_DN | 0.3835 | 0.6272 | 0.9252 |
| __JAK2_DN.V1_UP | 0.0675 | 0.8674 | 0.9394 |
| __KRAS.600.LUNG.BREAST_UP.V1_DN | 0.4484 | 0.4825 | 0.9923 |
| _HE | 0.6469 | 0.7549 | 0.5569 |
| __STK33_DN | 0.0987 | 0.8288 | 0.9526 |
| _HU | 0.3987 | 0.8766 | 0.7008 |
| __PRC2_EED_UP.V1_UP | 0.9742 | 0.6603 | 0.4371 |
| __SRC_UP.V1_UP | 0.7813 | 0.7356 | 0.5215 |
| __KRAS.LUNG.BREAST_UP.V1_UP | 0.9187 | 0.1144 | 0.9342 |
| __IL2_UP.V1_DN | 0.2528 | 0.6702 | 0.9829 |
| _BEN-PORATH-PRC2 | 0.6736 | 0.4381 | 0.8936 |
| _REUTER | 0.5337 | 0.8193 | 0.6846 |
| __PRC1_BMI_UP.V1_UP | 0.0878 | 0.8927 | 0.9493 |
| __KRAS.KIDNEY_UP.V1_UP | 0.4108 | 0.6545 | 0.9295 |
| _VANTVEER | 0.2137 | 0.8201 | 0.9276 |
| __KRAS.600_UP.V1_DN | 0.5252 | 0.4755 | 0.991 |
| __ATF2_UP.V1_UP | 0.8854 | 0.7283 | 0.5285 |
| __CAMP_UP.V1_UP | 0.7862 | 0.4271 | 0.8857 |
| __MEL18_DN.V1_DN | 0.5523 | 0.9608 | 0.5383 |
| _HALLSTROM | 0.4471 | 0.6143 | 0.9983 |
| __PDGF_UP.V1_DN | 0.7151 | 0.7473 | 0.7135 |
| __ESC_V6.5_UP_LATE.V1_UP | 0.3515 | 0.9714 | 0.7693 |
| LALONDE | 0.7414 | 0.7585 | 0.6745 |
| __PRC2_EZH2_UP.V1_UP | 0.6843 | 0.4385 | 0.9785 |
| __P53_DN.V1_UP | 0.6977 | 0.5957 | 0.8477 |
| __HOXA9_DN.V1_DN | 0.4022 | 0.8021 | 0.9261 |
| __CYCLIN_D1_UP.V1_UP | 0.6113 | 0.6402 | 0.9005 |
| __KRAS.BREAST_UP.V1_UP | 0.5307 | 0.5828 | 0.9992 |
| __ALK_DN.V1_UP | 0.8853 | 0.9537 | 0.4095 |
| __KRAS.DF.V1_DN | 0.9196 | 0.861 | 0.466 |
| __ATM_DN.V1_UP | 0.8797 | 0.8228 | 0.5373 |
| __CTIP_DN.V1_UP | 0.8849 | 0.6195 | 0.7756 |
| __NFE2L2.V2 | 0.5031 | 0.8984 | 0.7831 |
| __SNF5_DN.V1_DN | 0.4934 | 0.965 | 0.757 |
| _MORI | 0.8916 | 0.6694 | 0.7396 |
| __YAP1_DN | 0.5016 | 0.9339 | 0.7837 |
| __NOTCH_DN.V1_DN | 0.5673 | 0.8459 | 0.7989 |
| __EGFR_UP.V1_DN | 0.962 | 0.6928 | 0.7144 |
| __SRC_UP.V1_DN | 0.7518 | 0.9861 | 0.5437 |
| __P53_DN.V1_DN | 0.692 | 0.8645 | 0.7558 |
| __PRC2_EED_UP.V1_DN | 0.7937 | 0.9515 | 0.6193 |
| __PRC2_SUZ12_UP.V1_UP | 0.6841 | 0.9335 | 0.736 |
| __PTEN_DN.V1_UP | 0.908 | 0.978 | 0.577 |
| __KRAS.LUNG.BREAST_UP.V1_DN | 0.7278 | 0.8051 | 0.8768 |
| __KRAS.KIDNEY_UP.V1_DN | 0.829 | 0.6259 | 0.9644 |
| __ESC_V6.5_UP_EARLY.V1_UP | 0.958 | 0.7834 | 0.7698 |
| __KRAS.50_UP.V1_UP | 0.7772 | 0.6439 | 0.9855 |
| __RAPA_EARLY_UP.V1_DN | 0.9924 | 0.7054 | 0.8318 |
| __RAPA_EARLY_UP.V1_UP | 0.8528 | 0.7456 | 0.9128 |
| __KRAS.PROSTATE_UP.V1_UP | 0.7693 | 0.8606 | 0.8158 |
| __JNK_DN.V1_DN | 0.7267 | 0.7558 | 0.9461 |
| __JNK_DN.V1_UP | 0.7065 | 0.7595 | 0.9853 |
| __RPS14_DN.V1_UP | 0.9806 | 0.7721 | 0.816 |
| __DCA_UP.V1_UP | 0.9995 | 0.6436 | 0.9205 |
| __ATF2_S_UP.V1_UP | 0.5817 | 0.9729 | 0.931 |
| __KRAS.600_UP.V1_UP | 0.8897 | 0.823 | 0.8489 |
| _WEST | 0.9438 | 0.8125 | 0.8655 |
| __KRAS.300_UP.V1_UP | 0.6931 | 0.8686 | 0.945 |
| __MTOR_UP.V1_UP | 0.5833 | 0.9781 | 0.9542 |
| __PTEN_DN.V1_DN | 0.9464 | 0.9215 | 0.9325 |

**Table S7. Primers and TMEFF2 shRNA targets used in this study.**

**PRIMERS FOR qRTPCR**

**Gene** **Forward sequence Reverse sequence**

BUB1B TGCTTCACCCTTCAGGATCT CACCTTGGACTCAAGTCACC

CDC45 CATGACAGCCTGTGCAACAC GGGAAGACCCATGTCTGCAA

CDK1 TCATCTCAGTCCTTATGGCAGT TGGCAAGAAACTGATGAGAACA

CENPI AGGAAAGCCCAGAAGAAAGG GCCTTGTAACCCCTGTGAAA

CLSPN AGAATGCCAGTCGCCCTATG CCTGTTGAGCACTTCCTGGT

ERCC6L GTGCAGATCCTGAAGTTATGCT CCCAAAGAATCCAATTATGGG

EXO1 CTGCAGAGTTCAAATGCATCA CGTAGCTTGGAGGTCTGGTC

NCAPG AGGGGTGTAAAAGCAACCCA CTGACACCTCCTGTTCGTCC

NUSAP1 GCTGGTCTCAAACTCCACCA GCTCCACTGAGGTGAAGGAA

RAD51 AGGTTTCTGCGGATGCTTCT GCAAACATCGCTGCTCCATC

RRM2 ACACAAACCATCGGAGGAGAG TCCCAATGAGCTTCACAGGC

Calnexin CCACTGCTCCTCCTTCATCTCC TTCCTCTACCTCCCACTTTCCATC

| **shRNA TARGET SEQUENCES IN TMEFF2** |
| --- |
| shTMEFF2-0: CCTTGCATTTGTGGTAATCTA |
| shTMEFF2-1: CGTCTGTCAGTTCAAGTGCAA |
| shTMEFF2-2: GCGCTTCTGATGGGAAATCTT |
